# A Xanthine Derivative With Novel Heat Shock Protein 90‐Alpha Inhibitory and Senolytic Properties

**DOI:** 10.1111/acel.70047

**Published:** 2025-03-17

**Authors:** Sandra Atlante, Luca Cis, Davide Pirolli, Michela Gottardi Zamperla, Veronica Barbi, Antonello Mai, Clemens Zwergel, Serena Marcozzi, Maria Elisa Giuliani, Giorgia Bigossi, Giovanni Lai, Fiorenza Orlando, Robertina Giacconi, Fabrizia Lattanzio, Giulia Matacchione, Chiara Giordani, Massimo Bracci, Fabiola Olivieri, Federico Boschi, Paola Tabarelli De Fatis, Giovanni Battista Ivaldi, Marco Malavolta, Antonella Farsetti, Maria Cristina De Rosa, Carlo Gaetano

**Affiliations:** ^1^ Laboratory of Epigenetics Istituti Clinici Scientifici Maugeri IRCCS Pavia Italy; ^2^ National Research Council (CNR)‐IASI “A. Ruberti” Rome Italy; ^3^ Università Cattolica del Sacro Cuore Rome Italy; ^4^ Institute of Chemical Sciences and Technologies “Giulio Natta” (SCITEC)—CNR Rome Italy; ^5^ Department of Drug Chemistry and Technologies Sapienza University of Rome Rome Italy; ^6^ Advanced Technology Center for Aging Research and Geriatric Mouse Clinic IRCCS INRCA Ancona Italy; ^7^ Scientific Direction IRCCS INRCA Ancona Italy; ^8^ Clinic of Laboratory and Precision Medicine IRCCS INRCA Ancona Italy; ^9^ Department of Clinical and Molecular Sciences, Occupational Medicine Polytechnic University of Marche Ancona Italy; ^10^ Advanced Technology Center for Aging Research IRCCS INRCA Ancona Italy; ^11^ Department of Clinical and Molecular Sciences (DISCLIMO) Università Politecnica Delle Marche Ancona Italy; ^12^ Department of Engineering for Innovation Medicine University of Verona Verona Italy; ^13^ Department of Radiation Oncology Istituti Clinici Scientifici Maugeri IRCCS Pavia Italy

**Keywords:** aging, *drosophila*, HSP90α inhibitors, senescence, senolytics, xanthine

## Abstract

The accumulation of senescent cells contributes to aging and related diseases; therefore, discovering safe senolytic agents—compounds that selectively eliminate senescent cells—is a critical priority. Heat shock protein 90 (HSP90) inhibitors (HSP90i), traditionally investigated for cancer treatment, have shown potential as senolytic agents. However, inhibitors face formulation, toxicity, and cost challenges. To overcome these limitations, we employed a virtual screening approach combining structure‐based prefiltering with a ligand‐based pharmacophore model to identify novel, potentially safe HSP90 alpha isoform inhibitors exhibiting senolytic properties. This strategy identified 14 candidate molecules evaluated for senolytic activity in primary human fetal pulmonary fibroblasts. Four compounds exhibited significant HSP90i and senolytic activity, including two novel compounds, namely K4 and K5. The latter, 1‐benzyl‐3‐(2‐methylphenyl)‐3,7‐dihydro‐1H‐purine‐2,6‐dione, structurally related to the xanthinic family, emerged as a promising, well‐tolerated senolytic agent. K5 demonstrated senolytic activity across various cellular senescence models, including human fibroblasts, mesenchymal stem cells, and breast cancer cells. It was also effective in vivo, extending lifespan in *Drosophila* and reducing senescence markers in geriatric mice. Additionally, the xanthinic nature of K5 implicates a multimodal action, now including the inhibition of HSP90α, that might enhance its efficacy and selectivity towards senescent cells, Senolytic index SI > 1320 for IMR90 cells, and SI > 770 for WI38 cells, underscoring its therapeutic potential. These findings advance senolytic therapy research, opening new avenues for safer interventions to combat age‐related inflammaging and diseases, including cancer, and possibly extend a healthy lifespan.

## Graphical abstract

This study aimed to find safe senolytic drugs that selectively eliminate senescent cells. Focusing on HSP90 inhibitors, we employed a virtual screening to identify novel, safer compounds (A). K5, a xanthinic candidate, effectively eliminated senescent cells in various cellular models (B), extended lifespan in flies, and reduced aging signs in mice (C). K5's safety profile and multimodal action highlight its therapeutic potential for combating aging and age‐related diseases. Created in BioRender. Spadotto, V. (2025) https://BioRender.com/u88m942.

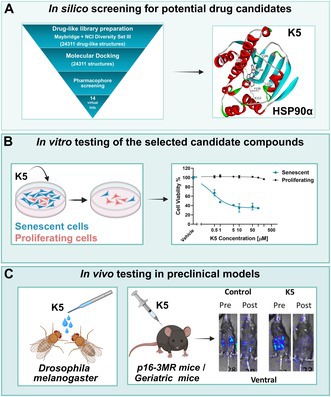

## 1 Introduction

The quest for senotherapeutics, interventions that selectively target or eliminate senescent cells, has gained significant momentum due to the recognition that these cells are pivotal in driving aging and age‐related diseases (McHugh and Gil 2018; Zhu et al. 2024). While several senolytic drugs, compounds designed to destroy senescent cells selectively, have shown promise, many are repurposed anticancer drugs with potential long‐term side effects (Mongelli et al. 2020). Additionally, some senolytics, like Dasatinib and Quercetin, have shown efficacy only in combination therapy (Hickson et al. 2019), limiting their applicability as standalone treatments. This evidence highlights a critical need for developing novel senolytic agents with improved safety profiles, targeted mechanisms of action, and the ability to function effectively as single agents.

Heat shock protein 90 (HSP90), a molecular chaperone overexpressed in many cancers and implicated in cellular senescence, has emerged as a compelling yet underdeveloped target for senolysis (Fuhrmann‐Stroissnigg et al. 2017). This highly conserved molecular chaperone system assists in the folding, maturing, and stabilizing of client proteins under normal and stressful conditions (van Oosten‐Hawle 2023). In mammals, the HSP90 family includes four major isoforms: HSP90α, which is cytoplasmic and stress‐induced, playing roles in cell signaling and survival pathways; HSP90β, also cytoplasmic but constitutively expressed, involved in housekeeping functions; TRAP1, found in mitochondria, crucial for maintaining mitochondrial health and regulating energy production; and GRP94, located in the endoplasmic reticulum, vital for the folding and processing of secreted and membrane‐bound proteins. Despite their structural similarity, these isoforms exhibit differences in client protein preferences and cellular functions (Sreedhar et al. 2004). HSP90 inhibitors have also been reported as a promising new class of senolytic drugs (Fuhrmann‐Stroissnigg et al. 2017). Thus, identifying HSP90 inhibitors represents a critical area of therapeutic development, leveraging their ability to disrupt multiple signaling pathways simultaneously by targeting a single chaperone protein essential for the function of numerous disease‐related proteins, including those essential for senescent cell survival (Fuhrmann‐Stroissnigg et al. 2018).

The toxicity of HSP90 inhibitors has been a significant concern in their development and clinical application, primarily due to the fundamental role of HSP90 in maintaining cellular homeostasis. Therefore, inhibiting HSP90 can lead to unintended consequences, affecting cancer and normal cells. Behind hepatotoxicity, the most common adverse reactions observed with HSP90 inhibitors include gastrointestinal disturbances and ocular toxicity. Hence, while HSP90 inhibitors hold great promise as therapeutic agents, their development is challenged by the need to balance efficacy with safety (Gedgaudas et al. 2024; Liu et al. 2024).

In this study, we undertook a virtual screening study to discover novel and safer HSP90 inhibitors, efficiently combining structure‐based (SBVS) and ligand‐based (LBVS) approaches (Sabe et al. 2021). Implementing this method, we identified 14 compounds tested in vitro to assess their ability to inhibit HSP90α function and induce senolysis. One of these, the synthetic xanthine derivative 1‐benzyl‐3‐(2‐methyl phenyl)‐3,7‐dihydro‐1H‐purine‐2,6‐dione, alias K5, progressed through in vivo studies to evaluate safety, effectiveness, and potential to mitigate age‐related conditions.

Synthetic xanthines are structurally similar to naturally occurring xanthines but have been chemically modified to enhance their pharmacological properties. One of the most critical features of synthetic xanthines is their potent bronchodilatory effect, which makes them valuable in treating respiratory conditions like asthma and chronic obstructive pulmonary disease (COPD). These compounds work by inhibiting phosphodiesterase enzymes, leading to increased cyclic AMP levels and subsequent relaxation of bronchial smooth muscle (Cazzola et al. 2018).

Another critical feature is their role as central nervous system stimulants. Synthetic xanthines can enhance alertness and cognitive function by blocking adenosine receptors, similar to Caffeine, though their effects are often more controlled and predictable due to their standardized dosages. Additionally, synthetic xanthines possess anti‐inflammatory properties, reducing inflammation in the airways and contributing to their therapeutic efficacy in respiratory diseases (Abu‐Hashem et al. 2024).

Moreover, xanthines like aminophylline are often formulated to improve solubility and bioavailability, ensuring more consistent and effective delivery of medical treatments. Their versatility extends to use in neonatal apnea, heart failure, and adjunctive therapy in other conditions requiring enhanced respiratory function (Monteiro et al. 2019).

This manuscript reports that a well‐tolerated synthetic xanthinic derivative with unprecedented HSP90α inhibitory properties displays senolytic activity in multiple cell models, including cancer cells; it also increases *Drosophila* lifespan and improves mice health span.

## 2 Materials and Methods

An extended Materials & Methods section is available as supplemental information, and a comprehensive list of detailed protocols and product specifications (Data S2).

### 2.1 Structure‐Based Virtual Screening

The screening generated a library of compounds complementary to the HSP90 ATP binding site. NCI Diversity Set III and the Maybridge HitFinder database were merged, filtered for drug‐likeness, and PAINS were removed. The remaining compounds were prepared using Schrödinger's LigPrep tool of Maestro to apply the OPLS5 force field. Epik was used to assign likely protonation states at pH 7.4 and tautomers. 24,311 structures underwent ligand‐flexible docking into the active site of 1YET at standard (SP) and extra precision (XP) levels. The top 50% from XP calculations were rescored with the Prime/MM‐GBSA method. The molecular docking algorithm was validated by redocking Geldanamycin into the HSP90‐ATP binding site and computing the RMSD between the heavy atoms of the ligand in the redocked pose and the crystallographic one.

### 2.2 Pharmacophore Modeling

The 3D virtual library was screened against a pharmacophore hypothesis based on known HSP90 inhibitors. LigandScout 4.1 was used to generate, refine, and screen the pharmacophore. Active compounds from the ChEMBL database with IC_50_ thresholds of 5 nM and 30 nm were used for the training and test sets, respectively. Excluded volumes were considered to improve model selectivity. The best‐fitting model was applied to the 3D virtual library representing the Glide solutions.

### 2.3 Cell Culture and Treatment

IMR90 and WI38 lung fibroblasts were purchased from ECACC‐Merck. Human bone marrow (BM)‐derived mesenchymal stromal cells (MSCs) were purchased from Lonza. Human umbilical vein endothelial cells (HUVECs) are primary cells obtained from a pool of donors purchased from Clonetics (Lonza). Murine ear fibroblasts (MearFs) were obtained from C57BL/6J mice maintained in the INRCA “Specific Pathogen Free” (SPF) animal facility. MCF7 breast cancer cells were purchased from ATCC. For dose–response curves, IMR90 and WI38 were induced to senescence through replicative passages; MSCs, HUVECs, and MearFs senescence was induced with Doxorubicin; MCF7 senescence was induced with 4‐Hydroxytamoxifen (Lee et al. 2014). The EC_50_ was calculated using GraphPad Prism 10 software. All treatment specifications are reported in the extended section.

### 2.4 Cell Viability Assay

Cell viability was determined using an MTT assay. Absorbance was measured using a NB‐12‐0035 Microplate Reader (NeoBiotech Co.). Data are expressed as a percentage of viability compared with untreated cells.

### 2.5 Senescence Associated‐β‐Galactosidase (SA‐β‐Gal) Colorimetric Assay

#### 2.5.1 For Cell Cultures

Previously treated cells were fixed according to the protocols established by the supplier (Cell Signaling). The images were acquired using an optical microscope (Leica) with 5× and 10× magnification (MAG) or the phase microscopy EVOS XL Core at 10× MAG and analyzed using the ImageJ software. The mean signal intensity was then normalized for the total number of cells.

#### 2.5.2 For Aged Tissues

Sections were prepared from organs and ear biopsies from snap‐frozen tissues and mounted on SuperFrost Plus slides (VWR). SA‐β‐gal staining was performed according to the manufacturer's instructions for the Sigma‐Aldrich‐QIA117 staining kit (Sigma‐Aldrich). Nuclei were counterstained with Nuclear Fast Red (Sigma‐Aldrich), and images were acquired using a Zeiss AxioCam HRc mounted on a Leitz Laborlux S light microscope. The percentage of senescent cells was determined with QuPath v. 0.3.2 (Bankhead et al. 2017).

### 2.6 HSP90 Inhibition Activity Assay

HSP90‐directed inhibitory activity was assessed employing the HSP90 N‐Terminal Domain Assay Kit (BPS Bioscience) according to the manufacturer's instructions. The fluorescence intensity was measured with a multiplate reader (Victor Nivo‐Perkin Elmer). The IC_50_ was calculated by analyzing the data using GraphPad Prism 10 software.

### 2.7 Cellular Thermal Shift Assay (CETSA)

CETSA was performed according to standard protocols (Jafari et al. 2014). Protein concentration was determined by the BCA assay (Life Technologies). Samples were analyzed using capillary electrophoresis Western blot automation (ProteinSimple, Biotechne) following the supplier's recommendations. Measurements were automatically calculated by the “Compass for SW” program.

### 2.8 Western Blot

WB was performed according to standard procedures and detected by UVITEC (Eppendorf). Densitometric analysis was performed with NIH ImageJ 1.8 software.

### 2.9 Immunofluorescence and Immunohistochemistry

Treated/untreated senescent IMR90 and MCF7 cells were fixed, permeabilized, and stained following suppliers' recommendations. Images were acquired using an Olympus IX83 research inverted microscope (IF) and a phase microscopy Nikon ECLIPSE Ei R (IHC) at 40× MAG.

### 2.10 Annexin V Staining

Senescent MCF7 cells were treated with K5 and Incucyte Annexin V NIR Dye (Sartorius). Cells were monitored using the Incucyte live‐cell Analysis system (Sartorius). Images were acquired using the phase microscopy EVOS XL Core at 10× magnification and analyzed using the ImageJ software.

### 2.11 Fluorescence Activated Cell Sorting (FACS)

Cells were treated with the 6‐chloromethyl‐2′,7′‐dichlorodihydrofluorescein diacetate, acetyl ester probe. Samples were analyzed by flow cytometry in the FITC channel using a FACS Melody (BD Biosciences).

### 2.12 Thermodynamic Solubility, In Vitro Metabolic Stability, and Permeability, K5 In Vivo Distribution After Oral (XOS) and Intravenous (IV) Administration

APHAD S.r.l (Buccinasco, MI, Italy) performed all evaluations. Detailed protocols are reported in the extended methods section.

### 2.13 *Drosophila melanogaster* Maintenance and Lifespan Assays

Experiments were carried out with wild‐type Canton‐S *Drosophila melanogaster*, originating (Stock #64349) from the Bloomington *Drosophila* Stock Centre (Indiana University). Flies were reared on Nutri‐Fly Bloomington Formulation food medium (Genesee Scientific). Vials containing only fresh food were alternated with vials containing food supplemented with K5. The number of living flies was counted every 2–3 days. Detailed protocols are reported in the extended section.

### 2.14 *Drosophila melanogaster* Gene Expression Analysis

Total RNA from *Drosophila melanogaster* was isolated using a TripleXtractor reagent kit (Grisp Research Solution, Porto, Portugal). Gene expression of *Dacapo (Dap), Unpaired 2 (Upd2), Unpaired 3 (Upd3), and Glyceraldehyde‐3‐phosphate dehydrogenase 1 (Gapdh1)* was analyzed by qRT‐PCR. Details are reported in the extended methods.

### 2.15 Mice and Experimental Design

All experiments were performed per the European Community Council Directives of 2010/63/UE. The pilot study (PS) investigated safety and potential senolytic efficacy, and the Extended study (ES) investigated functional changes, pathological phenotype, and the principal organ targeted by K5. Protocols were approved according to current Italian law (*D.Lgs*. n. 26/2014) by OPBA (animal care and health committee) of IRCCS INRCA and by the General Direction of Animal Health and Veterinary Drugs of the Italian Ministry of Health with authorization n°‐137/2021‐PR for PS and authorization n°‐370/2022‐PR for ES. In the PS of p16‐3MR mice (26 months), bioluminescence associated with p16‐expression was monitored before and after treatment, and the health of the mice was longitudinally studied. The ES used geriatric C57BL/6J mice (31 months). Clinical health and physical performance were analyzed before and after K5 treatment. Subsequently, the animals were euthanized, gross necropsies were performed, and organs were collected for further analysis. Detailed protocols are reported in the extended section.

### 2.16 Circulating Cytokines Assay and Biochemical Parameters Analysis

Blood samples were collected from the right retroorbital plexus. Glucose (GLUC), creatinine (CREA), aspartate aminotransferase (AST/GOT), and alanine aminotransferase (ALT/GPT) were determined in EDTA‐plasma by using an automated analyzer (Cobas Mira, Roche) according to the manufacturer's instructions. Cytokines were measured in EDTA‐plasma using the ProcartaPlex Mouse Cytokine & Chemokine Panel 1, 26plex (Thermofisher) and read in the Luminex 200 instrument (Bio‐Rad), following the producer's recommendation.

### 2.17 p21 and p53 mRNA Expression in Ear Biopsies

Total RNA was extracted from ear biopsies using the RNeasy kit (Qiagen) according to the manufacturer's instructions and quantified by NanoDrop. β‐actin, p‐21, and p‐53 gene expression levels were analyzed on a BioRad iQ5 optical RT‐PCR (BioRad).

### 2.18 Functional Phenotyping in Mice

We measured the clinical frailty index (CFI), Clinical Health Score (CHS), Physical Function Score (PFS), and Vitality Score (VS) in mice, as previously described (Marcozzi et al. 2023). All frailty measurements were performed in a dedicated INRCA SPF animal facility area. The CFI score for each mouse was calculated as previously published (Whitehead et al. 2014). The VS was obtained by calculating the arithmetic mean of the individual values of CHS and PFS. Several tests were assessed for endurance and speed Scores; details are reported in the extended methods.

### 2.19 “In Vivo” Bioluminescence Assay

Experimental animals' bioluminescence imaging (BLI) was performed with an IVIS Spectrum system (PerkinElmer). Mice were intraperitoneally injected with Xenolight RediJect Coelenterazine h (PerkinElmer), and bioluminescent images were obtained with mice in the dorsal and ventral positions; the Living Image software quantified bioluminescence acquired by the CCD camera.

## 3 Statistical Analysis

Kaplan–Meier (SPSS 26.0) estimated differential survival patterns with the Log‐Rank test. The chi‐square test was used to test the distribution of pathologies detected by gross necroscopy performed during organ explants or post‐mortem between treated and control groups. The other statistical analyses were performed using the GraphPad Prism 10 software. Student's *t*‐test, Wilcoxon, or Mann–Whitney *U* tests were used to compare typically paired non‐normally or independent non‐normally distributed data between two experimental groups. ANOVA and adequate *post hoc* tests were used for multiple comparisons. The General Linear Model with a univariate approach was employed to adjust for sex as a covariate. Data indicate the mean values of at least three independent experiments ± SD or SEM or the mean with 95% CI; sample sizes (*n*) and *p*‐values were reported in the corresponding figure legends. Outliers were identified and excluded using the ROUT method (Q = 1%) or Grubbs test (alpha = 0.05).

## 4 Results

### 4.1 Identification of Potential HSP90 Inhibitors With Senolytic Activity

**FIGURE 1:**
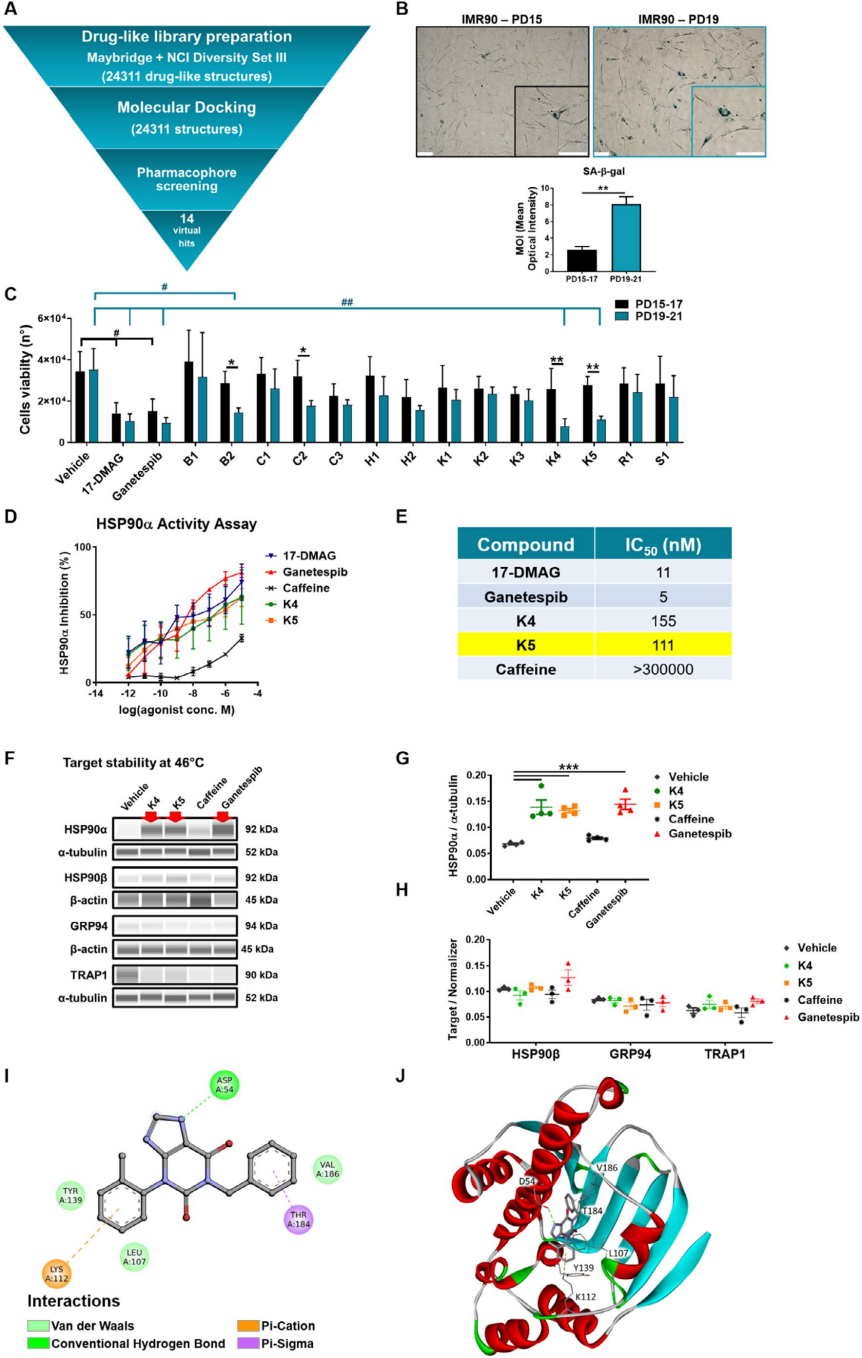
In vitro drug screening on IMR90 (Human fetal primary fibroblast) and target validation, K5 structure, and molecular interactions with HSP90. (A) Workflow diagram of the combined structure‐based and ligand‐based virtual screening strategy. (B) Representative images of SA‐β‐galactosidase staining on early‐passage cells (PD15, upper‐left) and advanced‐passage cells (PD19, upper‐right); 10× magnification, scale bar = 100 μm. Lower panel: The graph shows the SA‐β‐galactosidase product quantification evaluated comparing experiments on PD15‐PD17 (black bars) and PD19‐PD21 (blue bars) cells, normalizing to cells number; *n* = 6; mean ± SEM; data were analyzed by Mann–Whitney test; **p < 0.005. (C) The graph represents the average cell number of cells surviving treatment with the 14 in silico‐selected compounds on the two groups of cell populations PD15‐PD17 and PD19‐PD21, at 1 μM concentration, *n* = 3; mean ± SEM. Data were analyzed by 2way ANOVA and two‐stage linear step‐up procedure of Benjamini, Krieger, and Yekutieli; *p < 0.05; **p < 0.01, for paired comparisons; and 2way ANOVA, Dunnett's post hoc test for Vehicle comparisons among proliferating (black brackets) and senescent cells (blue brackets), ^#^ *p* < 0.05; ^##^ *p* < 0.005. (D) The graph represents HSP90‐α inhibition dose–response curves between 1 pM and 10 μM in a concentration range. (E) The table shows the extrapolated IC_50_; *n* = 3; mean ± SEM. (F) Representative images of HSP90 isoform stability were tested at 46°C in the presence of HSP90 inhibitors (0.5 mM). DMSO and Caffeine were used as solvent and negative controls, while Ganetespib was used as a positive control. α‐tubulin and β‐actin were used as normalizers. (G‐H) The graphs show the normalized protein levels; *n* ≥ 3; mean ± SEM. 1way ANOVA and Dunnett post hoc test analyzed data; **p < 0.005. (I) 2D interaction plot of K5 with the amino acid residues within the HSP90 binding pocket. The plot was generated using the Biovia Discovery Studio using three‐dimensional structural analysis of the HSP90‐ligand complex. (J) Solid ribbon representation of HSP90 bound to K5. Key interacting residues are depicted as stick models, colored by atom type. Biovia Discovery Studio was used to visualize 3D structures. Critical interactions between HSP90 and K5, identified using geometric and chemical criteria in the Discovery Studio Visualizer, are highlighted as dashed lines. Compound K5 shows a predicted binding free energy of −43.06 kcal/mol as calculated by the MM‐GBSA scoring approach and a Glide score of −4.883 kcal/mol.

A structure‐based virtual screening approach generated a focused library of high‐affinity compounds for HSP90α to be assayed against a ligand‐based pharmacophore model. Docking the crystallographic ligand to HSP90 was the first step in evaluating the molecular docking methodology and assessing the docking program and scoring function. The RMSD value between the redocked Geldanamycin structure and the crystallographic pose was 0.8 Å (Figure S1A), confirming the accuracy of our docking methodology (values < 2 Å are considered satisfactory). Figure 1A presents the general workflow of the multistep virtual screening approach implemented in this work.

After selecting druglike compounds, we employed a fast‐docking protocol to filter the NCI Diversity Set III and the Maybridge HitFinder libraries using the crystallographic structure of HSP90. Structure‐based virtual screening targeting the ATP‐binding site helped identify compounds with a high affinity for the chaperone protein. The final rank of each compound was determined by the binding free energy as calculated by MMGBSA. The generated focused library of the Top 80% solutions was then screened against the best pharmacophore hypothesis generated with LigandScout, one of the compound library enrichment algorithms with the best performance (Sanders et al. 2012).

The 14 compounds from the ChEMBL database with experimentally demonstrated inhibitory activity against HSP90 (IC_50_ threshold of 5 nM) were selected as a training set for generating the pharmacophore model. The result of the shared pharmacophore generation with LigandScout was a model with five H‐bond acceptors and three H‐bond donors, three aromatic rings, and two hydrophobic features, one positive ionizable feature, as well as 45 excluded volumes to account for the shape and boundary of the binding site. The model was further validated by a test set using a dataset of 73 known inhibitors from the ChEMBL database (IC_50_ threshold of 30 nM) and 1000 decoys to determine its level of ability to differentiate between active and inactive compounds. The generated pharmacophore hypothesis showed an area under the ROC curve of 0.73, shown in Figure S1B–S1C.

The pharmacophore‐based screening step identified 14 virtual hits (Table S1). They were chosen for in‐depth experimental evaluations to select those with better senolytic activity and lower toxicity on replicating cells. We set up the experimental conditions for the preliminary screening of HSP90 inhibitory compounds using the human fetal primary fibroblast cell line, IMR90, as the target cells. IMR90 cells were continuously culture‐expanded until about population doubling 19–21 (PD19‐21) when the cells reached replicative senescence (RS). The senescent phenotype (Figure 1B) was confirmed by the acquisition of the typical, enlarged, and flattened morphology of senescent cells (SCs) and the higher SA‐β‐galactosidase activity (SA‐β‐gal) in PD19 cells compared to early‐passage, non‐senescent cells (PD15).

To identify which compounds could be senolytic candidates, both PD15 and PD19 cells were exposed to the 14 candidate compounds (1 μM), and cellular viability was assessed after 48 h. Data indicated that four of these compounds showed selective cytotoxicity towards SCs. Among them, K4 and K5 exhibit the highest senolytic activity on SCs (Figure 1C, blue bars) while concurrently maintaining a robust level of safety for non‐senescent cells when compared to reference HSP90i compounds, 17‐DMAG and Ganetespib that, at the same concentration, exert cytotoxic effects also visible in non‐senescent cells (Figure 1C, black bars).

The selected compounds, K4 and K5, were further evaluated for their inhibitory activity on HSP90. Analysis of their in vitro inhibitory activity against HSP90α demonstrated a dose‐dependent inhibition for both compounds, with K5 exhibiting a higher potency (IC_50_ = 111 nM) compared to K4 (IC_50_ = 155 nM) (Figure 1D,E). These results were compared with those of the standard drugs 17‐DMAG and Ganetespib as positive controls, which showed IC_50_ values of 11 nM and 5 nM, respectively, while Caffeine was the negative control (IC_50_ > 300,000).

To further evaluate in vitro the direct interaction between compounds K4 and K5 and the HSP90 family isoforms, we performed a thermal shift assay on cellular lysates (CETSA), validating indeed the potent and selective interaction between our compounds and the HSP90α isoform. Whereas, under the same conditions, we did not observe a stabilization of the HSP90β isoform, the organ‐specific GRP94 (a 94 kDa glucose‐regulated protein found in the endoplasmic reticulum) and TRAP1 (tumor necrosis factor (TNF) receptor‐associated protein 1 located in the mitochondrial matrix). Caffeine was used as a negative control and does not interact with the protein in this condition, while Ganetespib was used as a positive control (Figure 1F–H).

Furthermore, the thermodynamic solubility in PBS of these compounds, their permeability in Caco‐2 cells, and metabolic stability against murine and human liver microsomes were evaluated. Results obtained in the solubility assay are reported in Figure S2A. Thermodynamic solubility is about 400 μg/mL for K4 and 200 μg/mL for K5. The metabolic stability of K4 and K5 was evaluated after incubation with mouse and human liver microsomes. Clearance data of the compounds and standards are reported in Figure S2B. Both compounds were stable in human microsomes and had a medium clearance in mice. Additionally, to evaluate the bidirectional permeability of the two compounds, we exploited Caco‐2 cells, an artificial model equivalent to the intestinal epithelium due to its physical and biochemical characteristics, which are pharmacologically ideal for evaluating ion and molecule passage through passive diffusion or active transport. The Trans Epithelial Electric Resistance (TERR) values at the beginning of the experiment were > 1000 Ωcm^2^, and at the end of the experiment, lucifer yellow permeability was null in all the wells. K5 showed excellent permeability in both directions. Whereas, probably due to a K4 solubility problem in the HBSS buffer, no K4 signal was detected in the apical or basolateral buffers (Figure S2C). In all tests performed, K5 outperformed K4 for solubility and permeability.

Therefore, we investigated further K5 compound pharmacokinetics, oral bioavailability, and tissue distribution in a CD1 mouse model. K5 concentrations in plasma after intravenous (IV; 5 mg/kg) and oral (XOS; 30 mg/kg) administration are reported in Figure S2D; K5 was rapidly eliminated in plasma with a linear decay after IV administration (T ½ 13 min), showing a low–mid clearance (38 mL/min kg) and a high distribution volume. The product is rapidly absorbed after oral administration with a Cmax at 10 min of 2802 ng/mL. K5 was quantified in plasma after oral administration up to 24 h, showing a mean residence time (MRT) similar to T ½ of approximately 300 min and a bioavailability of 54%. We also evaluated K5 concentration in the brain and kidney after IV administration at 5 mg/kg. The product is highly distributed in the kidney (131%) and also in the brain (35%), showing in the tissue a similar linear decay as detected in plasma (Figure S2E).

These observations show that K5 is the most promising compound, warranting further investigation.

Compound K5, the 1‐benzyl‐3‐(2‐methylphenyl)‐3,7‐dihydro‐1*H*‐purine‐2,6‐dione, contains a xanthine scaffold with substituents at positions 1 and 3, represented by a benzyl group and a 2‐methylphenyl group, respectively. K5 is a small and moderately lipophilic molecule with a molecular weight of 332.36 g/mol and a predicted logP of 2.63. With a topological surface area (TPSA) of 72.68 Å^2^, three rotatable bonds, three h‐bond acceptors, and one h‐bond donor, it meets the requirements to be classified as a drug‐like molecule according to Lipinski, Veber, and Ghose rules (Ghose et al. 1999; Veber et al. 2002; Lipinski et al. 2001).

Non‐bonding interactions that anchor the compound to the HSP90 ATP binding site are shown in Figure 1I,J.

One pi‐sigma interaction is formed between the six‐membered ring of the purine moiety of K5 and the γ‐methyl group of HSP90 Thr184. On the opposite side, the phenyl ring at position 3 of the purine ring system establishes pi‐cation interactions with the charged side chain of Lys112. A hydrogen bond is formed between the carboxyl oxygen of Asp54 and the N7 atom of purine. Additional van der Waals interactions involve the non‐polar regions of the molecule and the side chains of residues Leu107, Tyr139, and Val186 (Figure 1I,J).

### 4.2 In Vitro Validation of K5 Senolytic Effects

To thoroughly evaluate the safety of K5 treatment, to investigate its potential senolytic effects, and to determine the effective dosage at which these senolytic effects become apparent, we set up an experiment employing IMR90 and WI38 fibroblasts, MSCs, HUVEC, a primary murine fibroblast cell line (MearF), and the MCF7 human breast cancer cell line as our targets.

**FIGURE 2:**
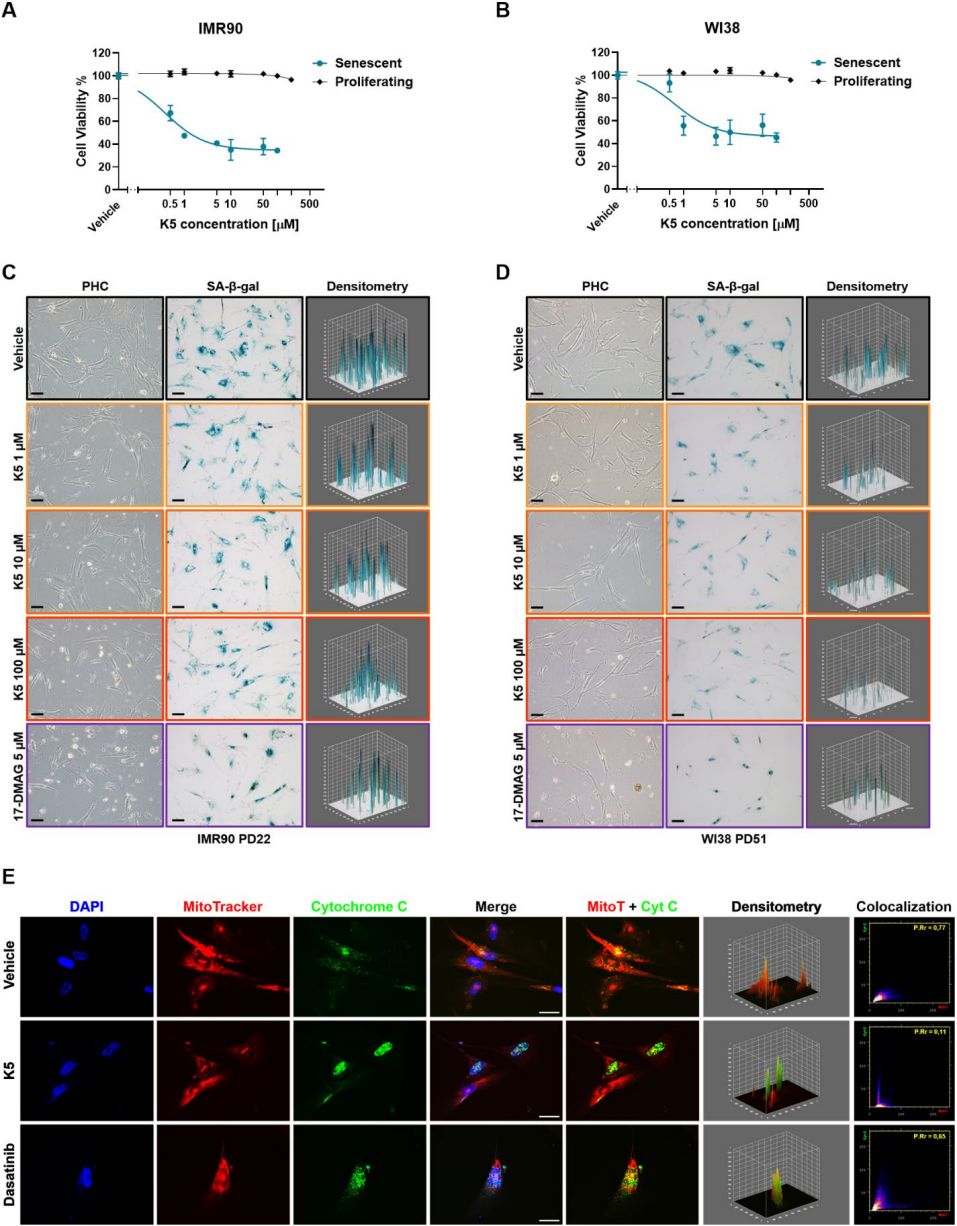
K5 senolytic effect on IMR90 and WI38 (Human fetal primary fibroblast). (A) Dose–response curves on proliferating (PD15, black line) and senescent (PD22, blue line) IMR90 cells treated with compound K5 at increasing concentrations (0.1, 0.5, 1, 5, 10, 50, 100 and 200 μM) for 48 h. (B) Dose–response curves on proliferating (PD29, black line) and senescent (PD51, blue line) WI38 cells treated with compound K5 at increasing concentrations (0.1, 0.5, 1, 5, 10, 50, 100 and 200 μM) for 48 h. Vehicle‐treated cells (DMSO) were used as solvent control. All cells were counted using Trypan blue reagent and EVAplus cell counter; data are presented as mean ± SEM, *n* = 3. (C, D) Phase contrast (PHC, left) and SA‐β‐galactosidase staining (center) representative images of senescent IMR90 (PD22, C) and WI38 cells (PD51, D), treated with K5 at 1 μM (yellow boxes), 10 μM (orange boxes), and 100 μM (red boxes), 17‐DMAG at 5 μM as positive control (purple boxes), and Vehicle as solvent control. Images were acquired at 10× magnification, scale bar = 100 μm. The 3D plots on the right show the densitometry of the blue colorimetric signal of the SA‐β‐galactosidase product, analyzed by ImageJ software. (E) Immunofluorescence representative images showing the Cytochrome C localization (green signal) in senescent IMR90 cells (PD21) after 48 h treatment with K5 25 μM, Dasatinib 1 μM as positive control, and Vehicle as solvent control (DMSO). DAPI (blue signal) and MitoTracker orange (red signal) were used to visualize nuclei and mitochondria, respectively. Images were acquired at 40× magnification, scale bar = 50 μm. The densitometry and the colocalization scatter plots were analyzed by ImageJ using the 3D and CoFinder plugins, showing a Pearson's Rr (PRr) = 0.769 and Overlap R (OR) = 0.913 for cells treated with Vehicle, PRr = 0.109 and OR = 0.454 after K5 treatment, and PRr = 0.643 and OR = 0.639 for Dasatinib treated cells.

Specifically, IMR90 and WI38 cells were continuously culture‐expanded until about PD22 and PD51, respectively, to reach replicative senescence (RS). After reaching RS, confirmed by the increased SA‐β‐galactosidase activity (SA‐β‐gal), K5 dose–response curves were performed on both senescent and proliferating cells (IMR90 at PD15 and WI38 at PD29) between 100 nM and 200 μM concentration (Figure 2A–D). K5 showed a significant senolytic effect on SCs at 500 nM, with an EC_50_ of 380 nM on IMR90 and an EC_50_ of 650 nM on WI38. At the same time, no evident toxicity was observed in proliferating cells in both cell lines; therefore, with a high estimated Senolytic index (SI), defined as the ratio between the EC_50_ of control cells and the EC_50_ of senescent cells, in both models, a SI > 1320 for IMR90 cells and a SI > 770 for WI38 cells was observed (Table 1). Of note, 17‐DMAG and Ganetespib dose–response curves in IMR90 cells (Figure S3A,B) showed a senolytic effect with an EC_50_ in SCs of 85 nM (SI = 9.2) and 87 nM (SI = 11.9), respectively (Table 1). Nevertheless, they also showed a substantial cytotoxic effect in proliferating cells (EC_50_ = 785 nM for 17‐DMAG and EC_50_ = 1035 nM for Ganetespib), leading to a difference of more than two orders of magnitude between their SI and K5's SI (> 1320) in the IMR90 model. Furthermore, the apoptotic effect of the K5 compound in senescent IMR90 cells (PD21) was confirmed through immunofluorescent analysis of the Cytochrome C (Cyt C) delocalization from the mitochondria, as represented in Figure 2E. Analyzing Cyt C and mitochondria colocalization, the Pearson's Rr (PRr) coefficient measured by CoFinder (ImageJ) was significantly lower in K5‐treated cells (PRr = 0.109) compared to cells treated with vehicle alone (PRr = 0.769) and the senolytic compound‐control Dasatinib (PRr = 0.643) (Napolitano et al. 2014).

**TABLE 1:** Effects of K5 on different models of cellular senescence.

Model	Compound	Senescence induction	Senolytic evidence (no, partly/yes)	EC_50_ senescent cells	EC_50_ proliferating cells	Senolytic index (estimated) or other info
Human fetal lung fibroblast (IMR90)	K5	Replicative/passages	Yes	0.38 μM	> 500 μM	SI > 1320, 20% reduced SA‐β‐gal^+^ cells
Human fetal lung fibroblast (IMR90)	17‐DMAG	Replicative/passages	Yes	0.085 μM	0.78 μM	SI = 9.2
Human fetal lung fibroblast (IMR90)	Ganetespib	Replicative/passages	Yes	0.087 μM	1 μM	SI = 11.9
Human fetal lung fibroblast (WI38)	K5	Replicative/passages	Yes	0.65 μM	> 500 μM	SI > 770, 10% reduced SA‐β‐gal^+^ cells
Human breast adenocarcinoma cells (MCF7)	K5	4‐hydroxy‐tamoxifen	Yes	24 μM	167 μM	SI = 7, 30% reduced SA‐β‐gal and Increased mortality (2.5‐fold) of senescent cells
Human umbilical vein endothelial cell (HUVEC)	K5	DNA damage (Doxorubicin)	No	> 500 μM	> 500 μM	NA
Human mesenchymal stem cells (MSCs)	K5	DNA damage (Doxorubicin)	Partly	> 200 μM	> 500 μM	SI = 9.7
Mouse ear fibroblasts (MearF)	K5	DNA damage (Doxorubicin)	No	> 500 μM	> 500 μM	NA

*Note:* The table reports the EC_50_ calculated after the K5 dose–response curves on proliferating cells and after senescence induction through various methods in six in vitro models and of compounds 17‐DMAG and Ganetespib in the IMR90 cell line. The estimated Senolytic Index (SI) shown in the last column is calculated as SI = EC_50_ in proliferating cells/EC_50_ senescent cells. EC_50_ has been extrapolated using GraphPad Prism 10 software.

Meanwhile, MSCs, HUVEC, and MearF cells were induced into senescence through treatment with Doxorubicin (DOXO). Subsequently, DOXO‐induced SCs (DOXO‐SCs) and non‐senescent cells (non‐SCs) were exposed to increasing concentrations of K5 (1–500 μM), and cell viability was assessed through an MTT assay (data not shown; EC_50_ is reported in Table 1).

Of note, DOXO‐induced senescent MSCs appear more susceptible to cell death than non‐senescent MSCs when treated with K5 at doses exceeding 5 μM, with an estimated SI = 9.7. In contrast, we observed no discernible difference in the vitality of K5‐treated DOXO‐induced senescent HUVEC and MearF versus their respective non‐senescent proliferating controls.

**FIGURE 3:**
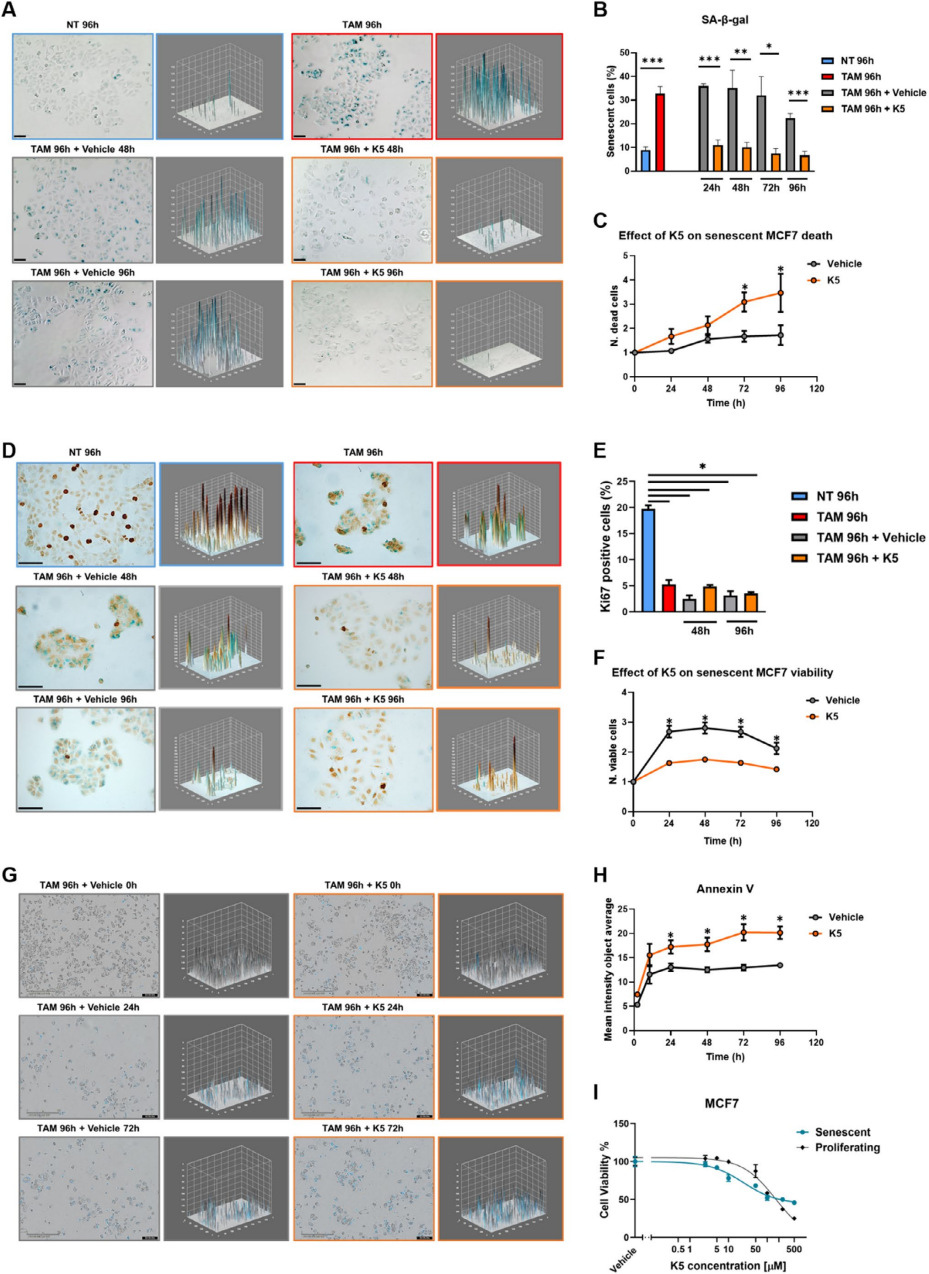
K5 senolytic effect on Tamoxifen‐induced senescent MCF7 breast cancer cells. (A, B) Representative images of SA‐β‐gal assay on MCF7 cells were obtained using the phase microscopy EVOS XL Core at 10× magnification and SA‐β‐gal assay performed on MCF7 cells after treatment with TAM (10 μM, 96 h) + K5 (10 μM, orange) or Vehicle (DMSO, gray) for 24 h, 48 h, 72 h, and 96 h (scale bar = 100 μm). The NT condition was treated with the TAM vehicle (EtOH 75%). Data plotted as the percentage of senescent cells (blue signal) over the total number of cells are represented as mean ± SEM of 5 independent experiments. Statistical significance was determined using a parametric paired two‐tailed Student's *t*‐test. *p < 0.05; **p < 0.01; ***p < 0.001. (C) Tamoxifen‐treated MCF7 cell mortality curves after treatment with K5 (10 μM, orange) or Vehicle (DMSO, gray) were performed using the Incucyte live‐cell Analysis system (Sartorius). Data plotted as the number of cells/well normalized to t0 (time of starting treatment) are represented as mean ± SEM of three independent experiments, each performed in quadruplicate. (D, E) Ki67 expression (dark brown) was evaluated by immunohistochemistry (IHC) upon Tamoxifen (10 μM, 96 h) + K5 (10μM) or Vehicle treatment (DMSO, 48 h and 96 h) in MCF7 cells—representative images obtained using the phase microscopy Nikon ECLIPSE Ei R at 40× magnification, scale bar = 50 μm. Data, plotted as the number of Ki67 positive cells (dark brown)/number of total cells expressed as a percentage, are represented as mean ± SEM of three independent experiments. Statistical significance was determined using a parametric paired two‐tailed Student's *t*‐test. *p < 0.05; **p < 0.01; ***p < 0.001. (F) Tamoxifen treated MCF7 cells viability curve after treatment with K5 (10 μM, orange), compared to Vehicle (DMSO, gray) performed using the Incucyte live‐cell Analysis system (Sartorius). Data, plotted as the number of cells/well normalized to t0 (time of starting treatment), are expressed as mean ± SEM of three independent experiments, each performed in quadruplicate. Statistical significance was determined using a parametric paired two‐tailed Student's *t*‐test; *p < 0.05. (G, H) Annexin V (blue signal) assay performed using the Incucyte live‐cell Analysis system (Sartorius) upon Tamoxifen (10 μM; 96 h) + K5 (10 μM) or Vehicle treatment (DMSO, over 96 h) in MCF7 cells—representative images obtained using the Incucyte (Sartorius, G), scale bar = 400 μm. (H) Data, plotted as the cells mean signal intensity (Annexin V positive cells, blue)/number of total cells, are represented as mean ± SEM of three independent experiments, each performed in quadruplicate. Statistical significance was determined using a parametric paired two‐tailed Student's *t*‐test. *p < 0.05. (I) Dose–response curves on proliferating (black line) and senescent (TAM, 10 μM, 96 h, blue line) MCF7 cells treated with compound K5 at increasing concentrations (2.5, 5, 10, 50, 100, 250, and 500 μM) for 96 h. Vehicle‐treated cells (DMSO) were used as solvent control—evaluation on proliferating or senescent MCF7 cells. The experiments were performed using the Incucyte live‐cell Analysis system (Sartorius). Data was plotted as a percentage of cell reduction vs control normalized to t0 (time of starting treatment).

Additionally, we induced senescence in a tumor cell line derived from hormone‐driven cancers, the MCF7 cell line, using a 4‐hydroxytamoxifen treatment (TAM, 10 μM, 96 h). The growth curve and SA‐β‐gal assay showed that TAM blocked MCF7 cell proliferation and induced senescence up to 35%–40% versus control (NT, Figure 3A,B). To evaluate the senolytic effect of compound K5, senescence‐induced cells were treated with the senolytic drug at 10 μM for a further 24 to 96 h (treatment with the senolytic drug was renewed at 48 h). SA‐β‐gal staining was drastically reduced, 10% vs 40% of senescent cells with K5 vs Vehicle (Figure 3A,B). Interestingly, compared to the control, the mortality rate of senescent cells detected over 96 h rose significantly with K5 (up to 2.5 folds, Figure 3C). We also evaluated the cell proliferation rate by analyzing Ki67 as a marker, which confirmed MCF7 cell cycle arrest by Tamoxifen and that K5 reduced senescent cells by its senolytic effect rather than a senomorphic one (Figure 3D,F). Furthermore, we evaluated the apoptotic effect of K5 in this model by analyzing Annexin V over 96 h treatment (Figure 3G,H), observing a significant and stable increase after 24 h in K5‐treated cells. Western blot analysis confirmed that TAM stabilized p21 and γH2AX, corroborating the senescence status and inducing DNA damage. Moreover, TAM diminished Cyclin D1, indicating cell cycle arrest, but its expression was revived after treatment with K5 or DMSO, while Caspase 3 further increased in K5‐treated cells compared to DMSO (Figure S3C,D). Additionally, we evaluated Reactive Oxygen Species (ROS) levels and Cytochrome C delocalization from mitochondria in MCF7, confirming the selective apoptotic effect of K5 in senescent cells, using as a positive control the Dasatinib compound (Figure S3E–G). Finally, also in this tumor model, we detected a K5 senolytic effect on senescent cells (EC_50_ = 24.4 μM) and low cytotoxicity in proliferating cells (EC_50_ = 167.2 μM), with a SI = 7 (Figure 3I and Table 1).

### 4.3 In Vivo Evaluation of K5 Effects

**FIGURE 4:**
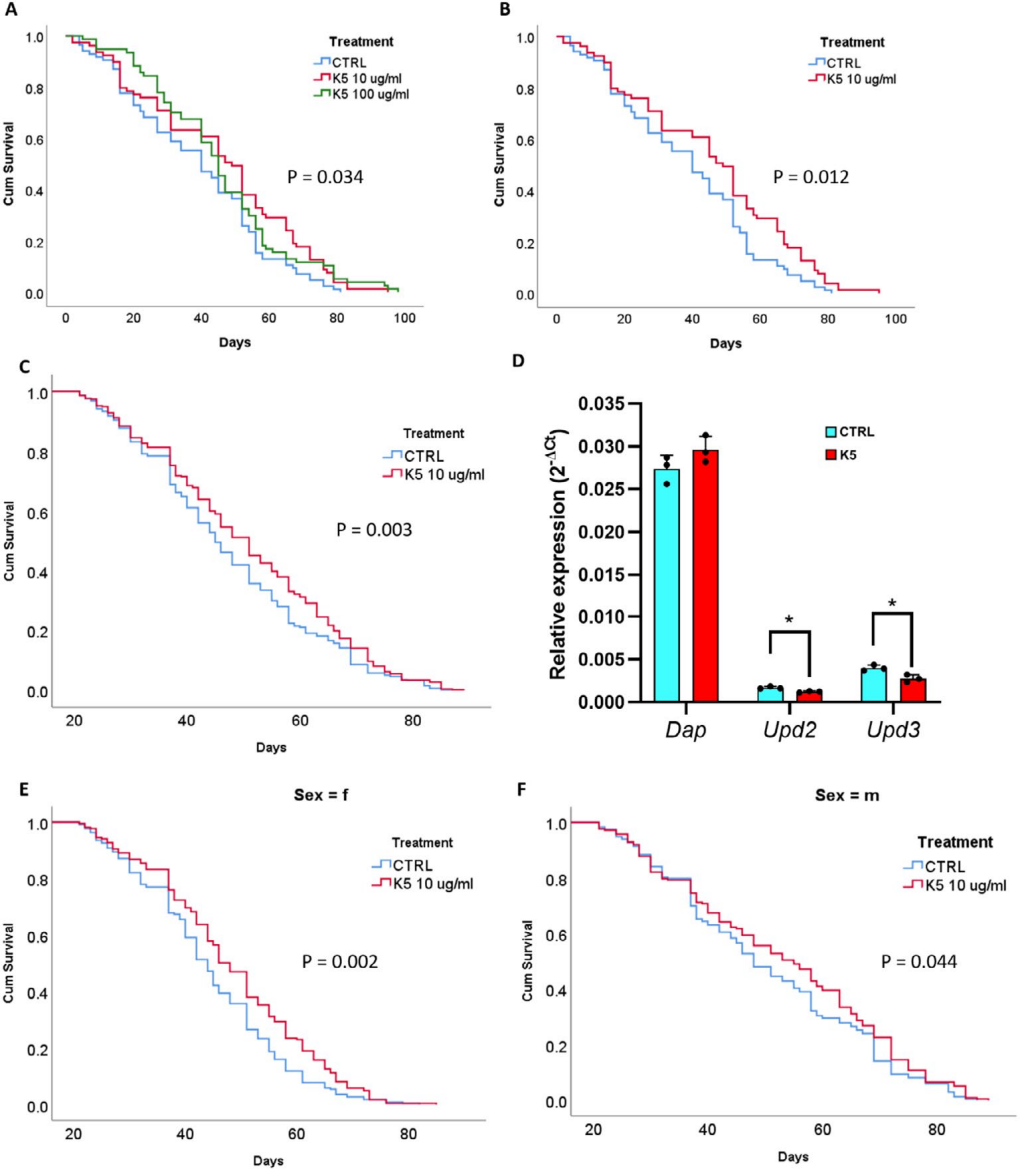
Effect of K5 on *Drosophila melanogaster* lifespan and gene expression of senescence‐related genes. (A) The panel shows the initial experiment testing two concentrations of K5 (10 μg/mL and 100 μg/mL) compared to control (CTRL) using 100 flies per group. Both concentrations showed improved survival, with a statistically significant effect (*p* = 0.034). Interestingly, the lower dose (10 μg/mL) appeared to perform better than the higher dose (100 μg/mL). (B) The graph represents the same data of panel A without the 100 μg/mL K5 group. Statistics are computed only by comparing the 10 μg/mL K5 group vs controls (CTRL). Here, the survival benefit becomes more evident, with K5‐treated flies showing consistently better survival throughout their lifespan (*p* = 0.012). The survival curves diverge, with K5‐treated flies maintaining higher survival rates than controls. (C) The panel presents an exciting finding in a larger population (*n* = 884, 445 males and 439 females) where K5 treatment (10 μg/mL) was started later in life (at day 20). Even with this delayed treatment start, K5 showed significant beneficial effects (*p* = 0.003). The data show that K5‐treated flies lived 50.69 days (±0.79 SE) on average, a 6.63% increase compared to control flies (47.54 days ± 0.74 SE). (D) The graph shows the relative expression levels (2^−ΔCt^) of *Dap*, *Upd2*, and *Upd3* in the K5‐treated group (10 μg/mL, red) and the control group (cyan). *Dap* expression was similar between groups, with no significant difference. In contrast, *Upd2* and *Upd3* expression levels were significantly lower in the K5‐treated group than in controls (*p < 0.05). Data represent means (±SD) from three independent experiments performed in triplicate, with individual means shown as single dots. (E, F) The graphs show the survival data from panel C, restricted to females (E, *p* = 0.002) and males (F, *p* = 0.044). These findings suggest that K5 extends lifespan when administered throughout life, reduces senescence markers, and provides benefits when treatment begins in mature flies. Notably, the effects were significant in both females and males, highlighting its potential therapeutic value even when intervention starts later in life.

To investigate the potential effects of K5 on *Drosophila* lifespan, we performed a preliminary treatment by administering two doses of K5 (10 μg/mL and 100 μg/mL) to 100 flies per group. The results revealed a significant overall difference in lifespan (*p* = 0.034; Figure 4A). Specifically, the lower dose (10 μg/mL) was responsible for the observed effect (*p* = 0.012; Figure 4B).

To investigate whether the effects of K5 are more pronounced when treatment is initiated later in a lifetime, in which the accumulation of senescent cells is more evident and to explore potential sex differences, we administered K5 starting at 20 days of age in a larger population of flies stratified by sex (*n* = 884; 445 males and 439 females, equally distributed between K5 and controls; Figure 4C). Despite the delayed start, K5 still conferred a significant survival benefit (*p* = 0.003). On average, K5‐treated flies lived 50.69 days (±0.79 SE), representing a 6.63% increase compared to controls (47.54 days ± 0.74 SE). When survival data were stratified by sex, the beneficial effect of K5 remained significant in both females (*p* = 0.002, Figure 4E) and males (*p* = 0.044, Figure 4F), demonstrating the robustness of the intervention across the sexes.

To explore potential mechanisms, we analyzed the relative expression levels of *Dap*, *Upd2*, and *Upd3* (Figure 4D). While *Dap* expression showed no significant difference between groups, the levels of *Upd2* and *Upd3* were significantly reduced in the K5‐treated flies compared to controls (*p* < 0.05), suggesting that K5 treatment affects the JAK/STAT signaling pathway, which may influence pathways associated with the inflammatory responses and senescence‐associated secretory phenotype (SASP).

Together, these findings indicate that K5 extends lifespan when administered throughout life and when initiated in adulthood, highlighting its potential therapeutic value for late‐onset intervention.

Further experiments were performed in mice. Considering that HSP90‐inhibiting compounds, such as 17‐DMAG, have been safely administered in mice within a range of 5–25 mg/kg (Park et al. 2020; Wang et al. 2016), we chose to test the effects of a dose of 10 mg/kg via i.p. injection in the pilot study (Figure S4). In this pilot study, K5 treatment did not induce any adverse reactions, observable behavioral stereotypes, or mortality events among the animals in the days following its administration.

It is worth noting that the median lifespan of treated animals in this pilot study was within the higher end of the typical range observed in our colony (K5 males: 1097 ± 78 days; K5 females: 953 ± 35 days). This effect is consistent with the higher lifespan ranges reported by other laboratories worldwide, reinforcing the observation that no toxic side effects were evident (Pabis et al. 2024).

**FIGURE 5:**
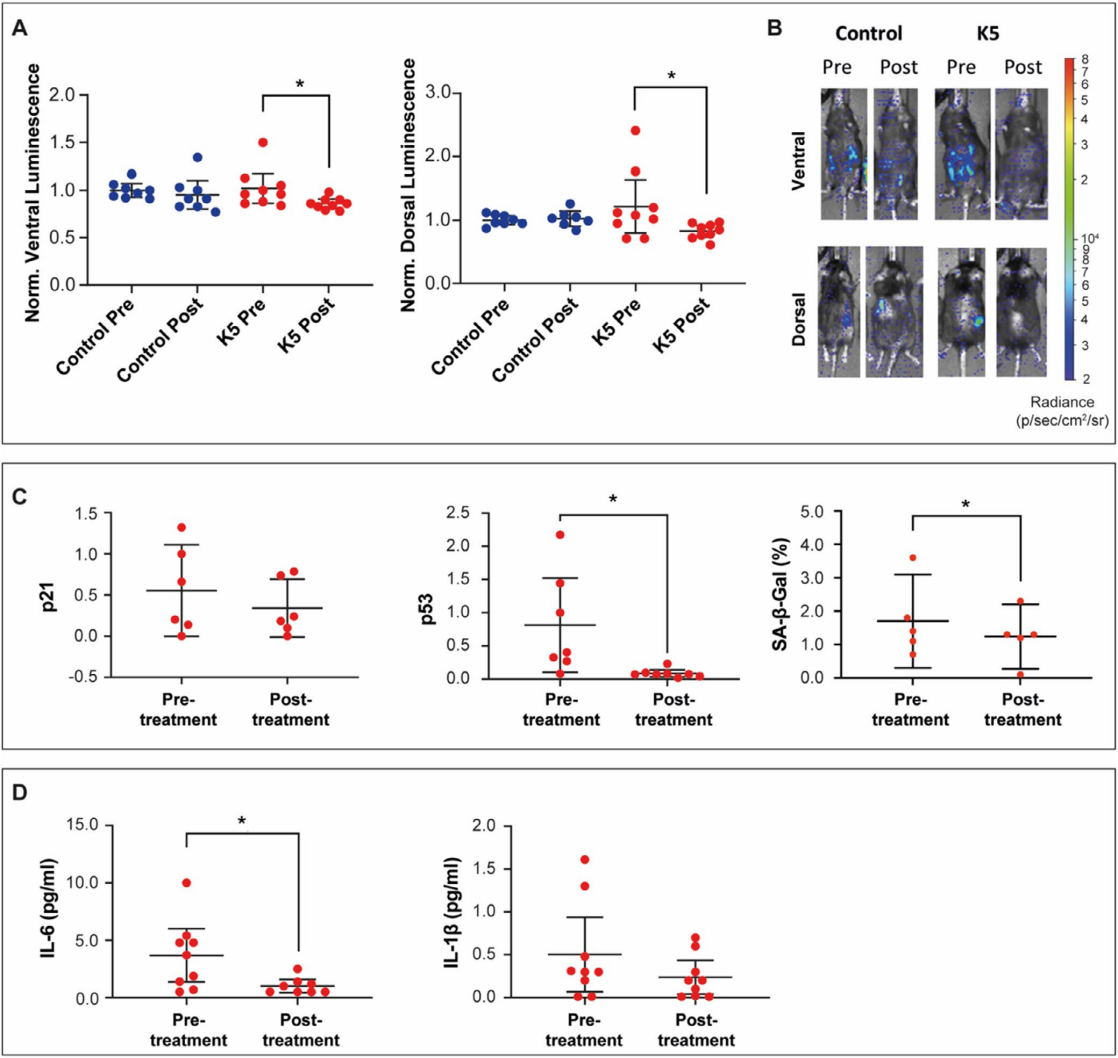
Live imaging and cellular senescence markers of p16‐3MR mice from the pilot study. (A) Average ventral (left) and dorsal (right) Luminescence of p16‐3MR mice treated with K5 (*n* = 9) compared to control p16‐3MR mice (*n* = 8) of the same age. All mice were treated at the age of 25 months. Data are expressed as mean (95% CI). *p < 0.05 by Wilcoxon test. (B) Representative images of p16‐3MR mice treated with K5 and untreated controls. (C) Expression of p21 and p53 and percentage of cells positive for SA‐β‐gal in ear biopsies of mice treated with K5 (*n* = 5–8). Data are shown as mean (95% CI). *p < 0.05 by Wilcoxon test. (D) Circulating levels of IL‐6 and IL‐1β in mice treated with K5 (*n* = 9; 5F and 4 M). Data are expressed as mean (95% CI). *p < 0.05 by Wilcoxon test.

The expression of p16, measured by in vivo bioluminescence assay, demonstrated a significant reduction in both ventral and dorsal signals at T1 when compared to T0 in K5‐treated mice (Figure 5A,B). These changes could indicate reduced senescent cell burden or differences in alopecia and coat conditions, which may affect luminescence detection in aging unshaved mice (Hoshino et al. 2017). Furthermore, we observed a substantial decrease in the expression of widely recognized senescence markers, that is, p21 and p53, as assessed by real‐time PCR on ear biopsy samples obtained from K5‐treated animals at T0 and T1. Notably, while p21 exhibited a decreasing trend, the reduction in p53 expression at T1 was statistically significant compared to baseline levels (Figure 5C). Additionally, K5 also led to a reduction in SA‐β‐gal activity in ear biopsies at T1 (Figure 5C).

In addition to molecular changes, K5 exerted a systemic impact, as evidenced by a reduction in circulating levels of interleukin‐6 (IL‐6) and a trend (*p* < 0.01) toward decreased interleukin‐1β (IL‐1β) at T1 compared to T0, two pro‐inflammatory cytokines associated with the secretory profile of SCs (Figure 5D).

**FIGURE 6:**
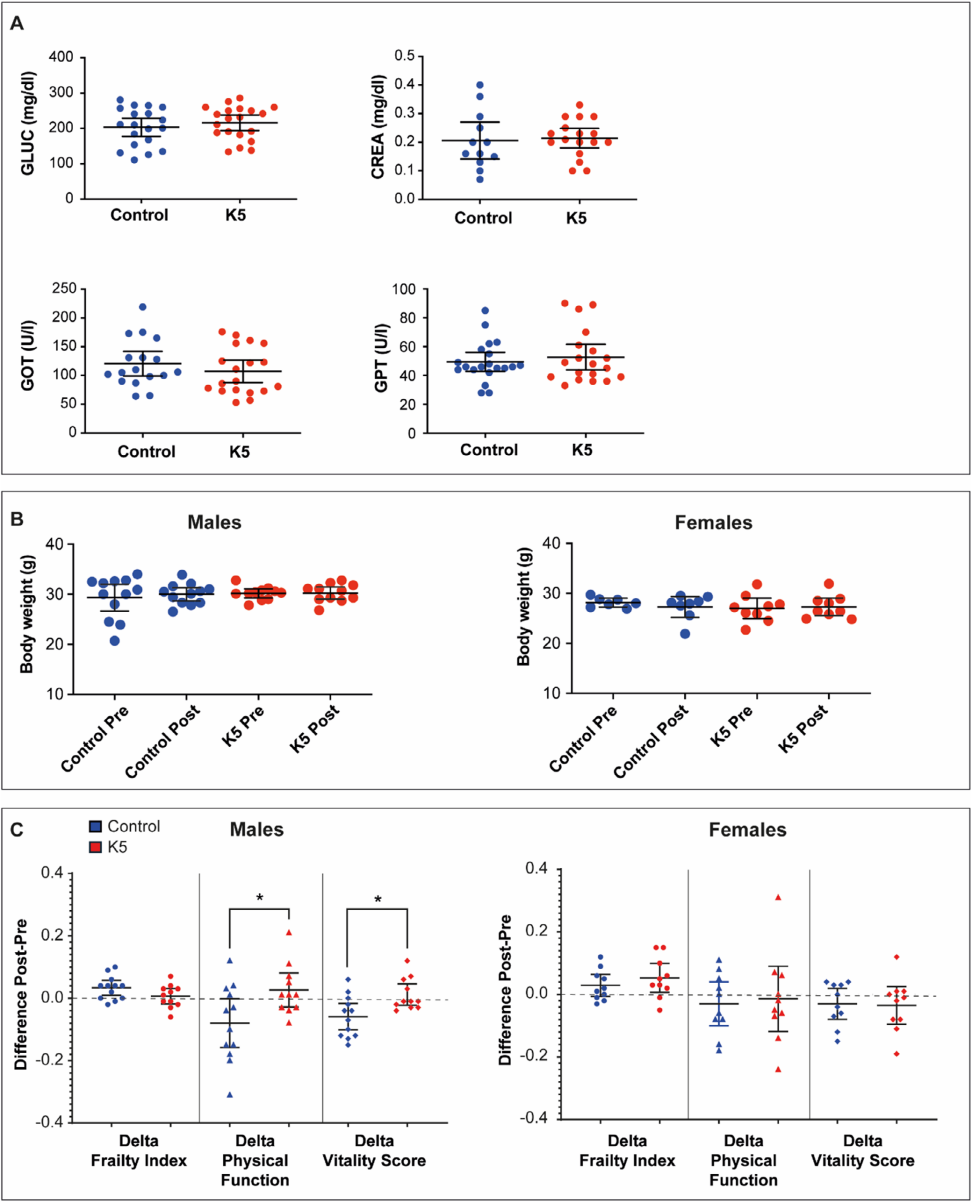
Clinical and functional status of geriatric mice (> 30 months) treated with K5. (A) Glucose, Creatinine, GOT, and GPT serum concentrations in treated mice compared to the control group. Data are expressed as mean (95% CI). (B) Body weight of male and female mice. Data are expressed as mean (95% CI). (C) Frailty Index, Physical function, and Vitality Score adjusted for baseline levels. Data are expressed as mean (95% CI). *p < 0.05 by Mann–Whitney *U* test.

**FIGURE 7:**
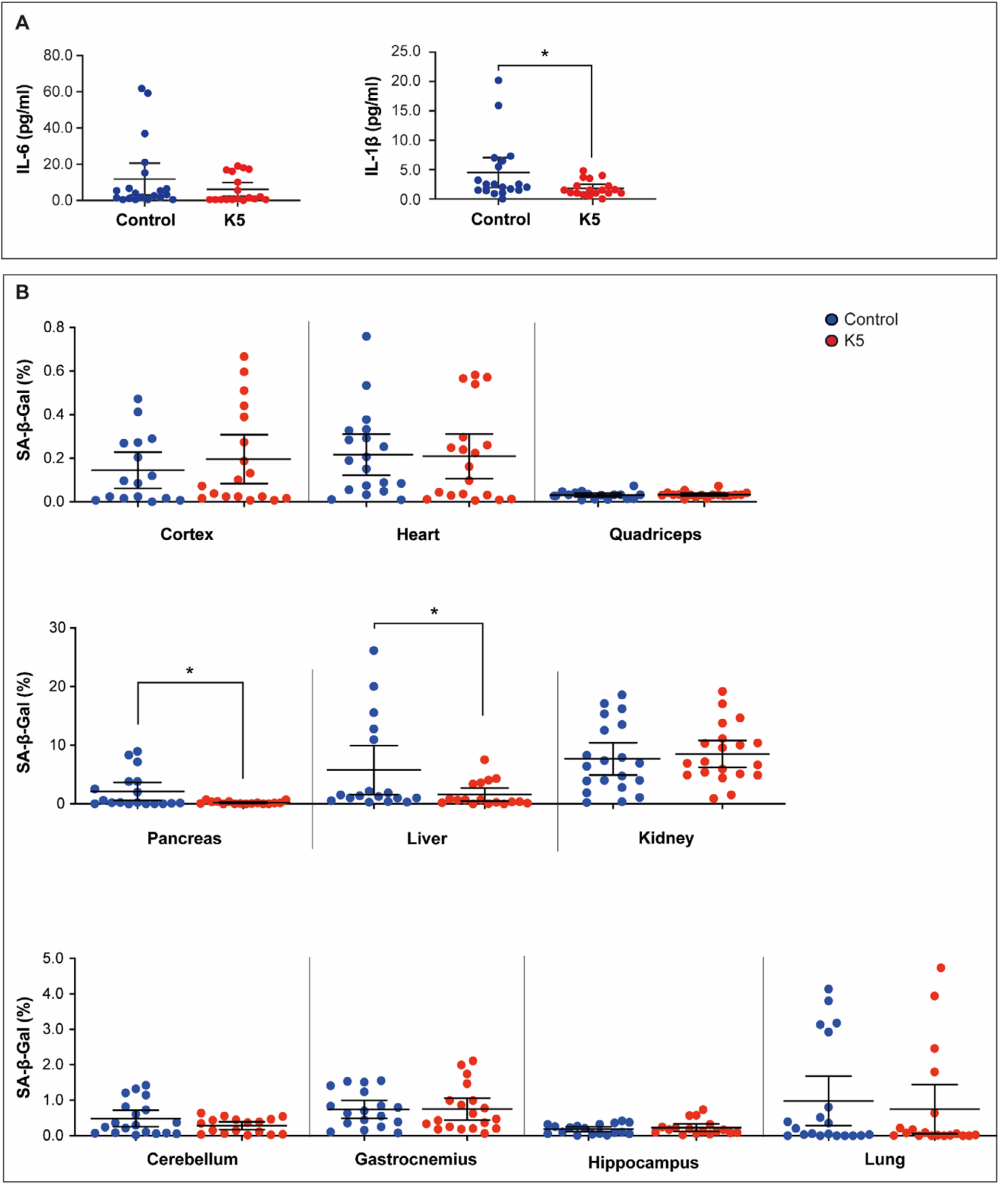
Cellular senescence markers in geriatric mice (> 30 months) treated with K5. (A) Circulating IL‐6 and IL‐1b levels in mice treated with K5 versus controls. Data are expressed as mean (95% CI). *p < 0.05 by General Linear Model (GLM) with a univariate approach, adjusting for sex as a covariate. (B) Multiorgan SA‐β‐gal assay showed as the mean % of SA‐β‐galactosidase positive cells (% SA‐β‐gal^+^ cells) of K5 (*n* = 20) and control (*n* = 20) mice analyzed in kidney, liver, lung, cortex, heart, quadriceps, cerebellum, pancreas, gastrocnemius, and hippocampus. Data are expressed as mean (95% CI). *p < 0.05 by General Linear Model (GLM) with a univariate approach, adjusting for sex as a covariate.

In the extended study (Figure S5), plasma levels of glucose, creatinine, GOT, and GPT showed no significant differences between treated and control groups, indicating the absence of systemic toxicity and the safety of K5 administration (Figure 6A). Consistently, body weight remained stable throughout the treatment period, further supporting the lack of adverse effects (Figure 6B). Interestingly, treated male mice demonstrated a significant increase in both the Physical Function Score and the Vitality Score compared to the control group, suggesting a positive effect of the treatment (Figure 6C). The analysis of pathology observed during gross necropsies revealed a lower incidence of tumors in treated male mice (Figure S6). Additionally, in the extended study, K5‐treated animals demonstrated a significant reduction in circulating IL‐1β levels, confirming an amelioration of systemic inflammation (Figure 7A). Finally, multiorgan SA‐β‐gal assay data highlight a significantly reduced staining in the liver and pancreas of K5‐treated mice compared to the control (Figure 7B).

## 5 Discussion

Heat shock protein 90 inhibitors have recently emerged as a promising class of senolytic agents capable of selectively inducing apoptosis in senescent cells. These cells accumulate with age and contribute to various age‐related diseases through the senescence‐associated secretory phenotype (SASP) (Fuhrmann‐Stroissnigg et al. 2017). HSP90, a molecular chaperone involved in stabilizing and properly folding numerous client proteins, is crucial for the survival of senescent cells by supporting anti‐apoptotic pathways. Inhibiting HSP90 disrupts these protective mechanisms, leading to the selective elimination of senescent cells while sparing normal, proliferating cells. This approach can extend the health span and offer therapeutic avenues for treating age‐related diseases (Dutta Gupta and Pan 2020; Fuhrmann‐Stroissnigg et al. 2018; Short et al. 2019). HSP90i is a miscellaneous class of compounds that typically share an ATP‐competitive binding site, which mimics the natural substrate of HSP90. This site often features a purine or purine‐like scaffold, such as in the cases of the purinic PU‐H71 and BIIB021 inhibitors, designed to fit into the ATP‐binding pocket of HSP90α. This interaction prevents ATP hydrolysis, inhibiting the chaperone activity and destabilizing its client proteins (He and Hu 2018).

In this direction, our study has relied on virtual screening to identify novel and safer HSP90α inhibitors with potential senolytic properties. While ligand‐based virtual screening (LBVS) does not require a 3D representation of the biological target, structure‐based virtual screening (SBVS) does (Feng et al. 2024; Macalino et al. 2015; De Donato et al. 2020). On the other hand, LBVS relies on the molecular similarity principle, which presents challenges with conformational flexibility and alignment, when molecules are represented and compared using their three‐dimensional properties (Berglund et al. 1997; Cramer et al. 2002). When information about the structure of ligand‐target complexes and similarity relationships to active compounds is available, combining SBVS and LBVS methods can improve the success of drug discovery studies (Debnath et al. 2019; De Donato et al. 2018). Here, a prefiltering structure‐based approach generated a focused library of high‐affinity compounds for HSP90 to be assayed against a ligand‐based pharmacophore model. From the initial screening, our protocol identified 14 compounds with potential HSP90 inhibitory activity (Table S1); four demonstrated evident senolytic activity in an in vitro assay. Finally, K4 and K5 emerged as novel compounds with unprecedented HSP90α inhibitory and senolytic properties (Figure 1C–E). Although K4 demonstrated promising senolytic activity and HSP90 binding in vitro (Figure 1F–G), a comprehensive characterization of its properties was hindered by its tendency to precipitate under certain solution conditions, such as in the presence of HBSS (not shown). This condition presented challenges in fully elucidating its properties. Despite these limitations, the ability of K4 to bind HSP90 and eliminate senescent cells warrants further investigation. Exploring alternative formulations or delivery systems may be necessary to improve K4 solubility and facilitate further studies.

However, K5, a xanthine‐based molecule, remained a promising senolytic agent in vitro and in vivo. It demonstrated enhanced selectivity towards senescent cells as evidenced by its Senolytic Indexes listed in Table 1. K5 also showed great stability and tissue distribution (Figure S2) and efficacy in reducing senescence‐associated markers (Figures 5, 6, 7).

Xanthines, a class of compounds including Caffeine, Theophylline, and Theobromine, have long been known for their multimodal pharmacological effects. The general structure of xanthines is characterized by a purine ring system consisting of a fused double ring comprising a six‐membered pyrimidine ring and a five‐membered imidazole ring, with two keto groups at positions 2 and 6. Functionally, they include purinergic receptor antagonism, where xanthines primarily act as antagonists of adenosine receptors. By blocking adenosine receptors, mainly A1 and A2A, xanthines reduce the effects of adenosine, such as drowsiness, sedation, and vasodilation. In addition, these molecules are known for their PDE inhibition: xanthines are well‐recognized inhibitors of phosphodiesterases (PDEs). PDE inhibition leads to an increase in intracellular levels of cyclic nucleotides like cAMP and cGMP. This modulation of intracellular signaling has significant anti‐inflammatory effects and applications in asthma, COPD, and inflammatory skin conditions (Zuo et al. 2019).

Interestingly, K5 belongs to a series of xanthines designed and synthesized to improve their PDE, particularly PDE4, inhibitory properties, reducing the efficacy on purinergic receptors and possibly the central nervous system side effects (Kyurkchieva and Baillie 2023; Montana et al. 1998). Indeed, K5 shares a core purinic structure similar to naturally occurring xanthines, like Caffeine and Theophylline. However, diverse functional groups are attached to the core structure, including a benzyl group linked to the nitrogen atom at position 1 of the purine ring and a 2‐methylphenyl group attached to the nitrogen atom at position 3. The combination of these functional groups and their arrangement differentiates K5 from naturally occurring xanthines, providing the molecule with a hydrophobic character that likely influences its specificity and lower toxicity.

Our research revealed that K5 is an effective and non‐toxic HSP90α inhibitor with senolytic properties. Xanthines and typical purinic HSP90 inhibitors share a purine‐based core, such as in BIIB021 or PU‐H71; however, the latter bear substituents at C8 and N9, whereas K5 modifies the N1 and N3. Nevertheless, synthetic xanthines influence enzyme activity (e.g., phosphodiesterase inhibition), leading to increased levels of cyclic AMP. HSP90 inhibitors, on the other hand, disrupt the function of HSP90 family, chaperones involved in properly folding and stabilizing client proteins, including many involved in cancer progression (Kudlova et al. 2022). Both synthetic xanthines and purinic HSP90 inhibitors can bind to ATP‐binding sites on proteins. For synthetic xanthines, this is relevant in inhibiting phosphodiesterase enzymes, while HSP90 inhibitors target the ATP‐binding site of the HSP90 protein, crucial for its chaperone activity.

The prediction analysis suggests that K5 benzyl and 2‐methyl phenyl groups could fit into the hydrophobic pocket of HSP90 (see Figure 1J), where their aromatic nature could facilitate π‐π stacking interactions as they occur for purinic HSP90α inhibitors. These computational docking studies have been supported by biochemical and biological assays that confirm that K5 can effectively bind and inhibit HSP90α, a property not shared by Caffeine, indicating that this function is not commonly present in the naturally occurring xanthines family. Although synthetic xanthines and purinic HSP90 inhibitors could bind to ATP‐binding sites on proteins, and this shared property might be the basis of the K5 effect, there is no particular similarity between the ATP pocket of PDEs and HSP90. Also, while our data show a direct binding between K5 and HSP90α (Figure 2F–G), we do not conclusively demonstrate that the senolytic activity is solely due to HSP90 binding, and possible off‐target effects are not ruled out.

Nevertheless, as a PDE (Montana et al. 1998) and HSP90α dual inhibitor, K5 may exert its beneficial effects through a combinatorial mechanism. PDE4 inhibition can suppress pro‐inflammatory pathways associated with SASP. In contrast, HSP90α inhibition likely disrupts the chaperone protective effects on pro‐survival client proteins in senescent cells, including AKT1 and other pro‐survival factors (Dutta Gupta and Pan 2020). This dual action makes K5 a compelling candidate for further investigation and, as the first of this kind, a promising lead compound to develop novel and even more effective derivatives.

Although, in *Drosophila*, cellular senescence follows pathways different from mammalian ones, the relevance of this model for investigating senolytics application in chronic diseases remains elevated (Miller et al. 2023). Remarkably, K5 contributed to lifespan extension in *Drosophila*, accompanied by reduced senescence markers, as evidenced by the decreased expression of *Upd2* and *Upd3* (Figure 4D). UPD2 and UPD3 are cytokines functionally analogous to mammalian IL‐6, and their reduction indicates reduced SASP‐associated inflammatory signaling (Ito and Igaki 2016).

This evidence agrees with a recent study showing pharmacological inhibition of HSP90 as a target to counteract aging in *C. elegans* (Janssens et al. 2019). The improved physical function, vitality score, and overall reduced incidence of tumors in treated male mice also suggest a potential antitumor activity of this compound. The multiorgan SA‐β‐gal assay data further corroborate these results, revealing a significant reduction in senescence‐associated β‐galactosidase staining in the liver and pancreas of K5‐treated mice, paralleled by decreased expression of SASP factors, such as IL‐6 and IL‐1β. Noteworthy, the impact of K5 on IL‐6 and IL‐1β seems to be age‐dependent as the mice in the pilot study (Figure 5D) were 5 months younger than those in the extended study (Figure 7A). The specific reduction in SA‐β‐gal staining in the liver and pancreas aligns with the observed lower incidence of tumors, suggesting that the senolytic agent may exert its effects by eliminating senescent cells, thereby reducing the overall tumor burden. Indeed, liver cancer of primary or secondary origin is among the most frequent causes of death in male C57BL/6J mice (Brayton et al. 2012).

Moreover, lymphoma can induce systemic effects on other organs, including the pancreas. Hence, K5 treatment might also mitigate the secondary effects of systemic diseases commonly affecting geriatric mice, like lymphoma. These findings suggest that K5's senolytic properties, combined with its favorable safety profile, hold the potential for applications in mitigating age‐related pathologies and promoting healthy aging.

While our study demonstrates the potential of K5 as a senolytic agent, the compound may also inhibit other HSP90 family members, and further investigation is needed to understand its long‐term effects and potential for clinical translation. The promising results in *Drosophila* and mouse models provide the foundation for future research. Still, it is crucial to acknowledge that these models may not fully recapitulate the complexity of human aging and age‐related diseases. Therefore, additional studies in diverse animal models and, eventually, human clinical trials are necessary to validate the efficacy and safety of K5 for therapeutic applications.

Specifically, future work should address the following aspects: (i) Determining the specific mechanisms by which K5 induces senolysis is crucial. In light of its multimodal mechanism of action, investigating its influence on crucial pro‐survival and inflammatory pathways involved in senescence will provide a deeper understanding of its mode of action; (ii) Exploring whether K5 can act synergistically with other known senolytic agents could open up possibilities for even more effective therapeutic strategies. This approach may optimize senescent cell elimination and overcome potential resistance mechanisms; (iii) A comprehensive pre‐clinical evaluation is necessary before exploring K5 in clinical trials for age‐related conditions. This analysis includes thorough investigations into its long‐term safety, optimal dosing strategies, and potential interactions with other medications.

In conclusion, our study reports the identification of an unprecedented xanthinic HSP90α inhibitor with senolytic activity. The discovery of K5, a safe and effective senolytic with potential multi‐target activity, contributes to the expanding field of senotherapeutics, laying the basis for developing innovative, safer therapeutics to combat age‐related diseases.

## Author Contributions

S.A., L.C., and D.P., conceived and carried out experiments and data analysis and revised the manuscript. D.P. carried out *in silico* work. M.G.Z., V.B., S.M., R.G., G.M., C.G., F.O., and P.T.D.F. performed in vitro experiments and revised the manuscript. M.E.G., G.B., G.L., F.O., M.B., and F.B. carried out in vivo experiments and revised the manuscript. A.M., C.Z., G.B.I., and F.L. revised data analysis and the manuscript and wrote grants. M.M. designed and obtained ethical approval for the mice study. S.A., S.M., A.F., M.C.D.R., M.M., and C.G. conceived and supervised experiments and wrote the manuscript. All the authors contributed critical discussion and approved the final version of the manuscript.

## Disclosure

All authors agreed to this publication.

## Conflicts of Interest

Prof. Carlo Gaetano and Drs. Sandra Atlante, Maria Cristina De Rosa, Davide Pirolli, Antonella Farsetti, and Marco Malavolta declare a conflicts of interest as part of the data presented in this study was used to file a patent (WO 2023/135533 A1) which has subsequently been granted to INRCA, CNR, and ICS Maugeri.

## Supporting information


Data S1.

Data S2.

## Data Availability

The datasets generated or analyzed during the current study are not publicly available due to their consideration in a patenting process but are available from the corresponding author on a reasonable request.
