# Supplementary material for: A Xanthine Derivative With Novel Heat Shock Protein 90‐Alpha Inhibitory and Senolytic Properties
Source: Aging Cell. 2025 Mar 17;24(7):e70047. doi: 10.1111/acel.70047 (PMC12266748; doi:10.1111/acel.70047)
Supplement: Supplementary file 2 — Data S2. [file ACEL-24-e70047-s001.docx]

**A Xanthine Derivative with Novel Heat Shock Protein 90-alpha Inhibitory and Senolytic Properties**

Sandra Atlante*^1,2^, Luca Cis*^3^, Davide Pirolli*^4^, Michela Gottardi Zamperla^1^, Veronica Barbi^1,3^, Antonello Mai^5^, Clemens Zwergel^6^, Serena Marcozzi^7^, Maria Elisa Giuliani^7^, Giorgia Bigossi^7^, Giovanni Lai^7^, Fiorenza Orlando^7^, Robertina Giacconi^7^, Fabrizia Lattanzio^8^, Giulia Matacchione^9^, Chiara Giordani^9^, Massimo Bracci^10^, Fabiola Olivieri^11,12^, Federico Boschi^13^, Paola Tabarelli De Fatis^14^, Giovanni Battista Ivaldi^14^, Marco Malavolta^§7^, Antonella Farsetti^§2^, Maria Cristina De Rosa^§4^, Carlo Gaetano^§1^.

1. Laboratory of Epigenetics, Istituti Clinici Scientifici Maugeri IRCCS, 27100, Pavia, Italy. [sandra.atlante@icsmaugeri.it](mailto:sandra.atlante@icsmaugeri.it), [michela.gottardizamperla@icsmaugeri.it](mailto:michela.gottardizamperla@icsmaugeri.it), [veronica.barbi@icsmaugeri.it](mailto:veronica.barbi@icsmaugeri.it), [carlo.gaetano@icsmaugeri.it](mailto:carlo.gaetano@icsmaugeri.it).
2. National Research Council (CNR)-IASI "A. Ruberti", 00185, Rome, Italy. [antonella.farsetti@cnr.it.](mailto:antonella.farsetti@cnr.it)
3. Università Cattolica del Sacro Cuore, 00168, Rome, Italy. [luca.cis@unicatt.it.](mailto:luca.cis@unicatt.it)
4. Institute of Chemical Sciences and Technologies "Giulio Natta" (SCITEC) - CNR, Rome, 00168, Italy. [mariacristina.derosa@cnr.it](mailto:mariacristina.derosa@cnr.it), [davide.pirolli@cnr.it](mailto:davide.pirolli@cnr.it).
5. Pasteur Institute, Cenci-Bolognetti Foundation, Sapienza University of Rome, 00185 Rome, Italy. [antonello.mai@uniroma1.it](mailto:antonello.mai@uniroma1.it).
6. Department of Drug Chemistry and Technologies, Sapienza University of Rome, Piazzale Aldo Moro 5, 00185 Rome, Italy. [clemens.zwergel@uniroma1.it](mailto:clemens.zwergel@uniroma1.it).
7. Advanced Technology Center for Aging Research and Geriatric Mouse Clinic, IRCCS INRCA, 60121, Ancona, Italy. [m.malavolta@inrca.it](mailto:m.malavolta@inrca.it), [s.marcozzi@inrca.it](mailto:S.MARCOZZI@inrca.it), [m.giuliani@inrca.it](mailto:M.GIULIANI@inrca.it), [g.bigossi@inrca.it](mailto:G.BIGOSSI@inrca.it), [g.lai@inrca.it](mailto:G.LAI@inrca.it), [f.orlando@inrca.it](mailto:F.ORLANDO@inrca.it), [r.giacconi@inrca.it](mailto:R.GIACCONI@inrca.i).
8. Scientific Direction, IRCCS INRCA, 60121, Ancona, Italy. [fabrizia.lattanzio@gmail.com.](mailto:fabrizia.lattanzio@gmail.com)
9. Clinic of Laboratory and Precision Medicine, IRCCS INRCA, 60127, Ancona, Italy. [g.matacchione@inrca.it](mailto:G.MATACCHIONE@inrca.it), [c.giordani@inrca.it](mailto:C.GIORDANI@inrca.it).
10. Occupational Medicine, Department of Clinical and Molecular Sciences, Polytechnic University of Marche, 60126 Ancona, Italy. [m.bracci@staff.univpm.it](mailto:m.bracci@staff.univpm.it).
11. Advanced Technology Center for Aging Research, IRCCS INRCA, Ancona 60121, Italy.
12. Department of Clinical and Molecular Sciences, Polytechnic University of Marche, Ancona 60126, Italy. [f.olivieri@staff.univpm.it](mailto:f.olivieri@staff.univpm.it).
13. Department of Engineering for Innovation Medicine, University of Verona, Strada Le Grazie 8, 37134, Verona, Italy. [federico.boschi@univr.it](mailto:federico.boschi@univr.it).
14. Department of Radiation Oncology, Istituti Clinici Scientifici Maugeri IRCCS, 27100, Pavia, Italy. [paola.tabarelli@icsmaugeri.it](mailto:paola.tabarelli@icsmaugeri.it), [giovannibattista.ivaldi@icsmaugeri.it](mailto:giovannibattista.ivaldi@icsmaugeri.it).

*Equal contribution.

§ Corresponding authors.

**Supplementary data**

## **Supplementary figures and tables**

| ***Compound*** | ***IUPAC name*** | ***Molecular formula*** | ***Structural formula*** | ***MW (g/mol)*** |
| --- | --- | --- | --- | --- |
| ***B1*** | *2,5-diethyl 3,4-dihydroxy-1-(4-methoxyphenyl)-1H-pyrrole-2,5-dicarboxylate* | *C17H19NO7* | ** | *349.339* |
| ***B2*** | *4-[4-(4-fluorophenoxy)-5-methyl-1H-pyrazol-3-yl]benzene-1,3-diol* | *C16H13FN2O3* | ** | *300.289* |
| ***C1*** | *1-(4-nitrophenyl)-5-phenyl-1H-1,2,3-triazole-4-carboxamide* | *C15H11N5O3* | ** | *309.285* |
| ***C2*** | *N-{[5-(2,4-difluorophenyl)furan-2-yl]methyl}benzenesulfonamide* | *C17H13F2NO3S* | ** | *349.350* |
| ***C3*** | *1-(2,4-dichlorobenzoyl)-5-[5-(2,4-difluorophenyl)furan-2-yl]-1H-pyrazole* | *C20H10Cl2F2N2O2* | ** | *419.210* |
| ***H1*** | *(2S,3S,4R,5S)-2-[6-amino-8-(dimethylamino)purin-9-yl]-5-(hydroxymethyl)oxolane-3,4-diol* | *C12H18N6O4* | ** | *310.314* |
| ***H2*** | *N-(1,3-benzodioxol-5-ylmethyl)-1-(4-methoxyphenyl)-5-methyl-1H-pyrazole-4-carboxamide* | *C20H19N3O4* | ** | *365.390* |
| ***K1*** | *methyl 4-[4-(2,4-dichlorophenyl)-1,3-thiazol-2-yl]-5-(methylsulfanyl)thiophene-2-carboxylate* | *C16H11Cl2NO2S3* | ** | *416.350* |
| ***K2*** | *5-bromo-1-(2-methylphenyl)-3-propylpyrimidine-2,4-dione* | *C14H15BrN2O2* | ** | *323.190* |
| ***K3*** | *2-(3-benzyl-1-isopropyl-2,4-dioxopyrimidin-5-yl)-1,3-thiazole-4-carbohydrazide* | *C18H19N5O3S* | ** | *385.440* |
| ***K4*** | *(Z)-N'-[(2,4-difluorobenzenesulfonyl)oxy]-2-(2-methylpropane-2-sulfonyl)ethanimidamide* | *C12H16F2N2O5S2* | ** | *370.390* |
| ***K5*** | *1-benzyl-3-(2-methylphenyl)-7H-purine-2,6-dione* | *C19H16N4O2* | ** | *332.363* |
| ***R1*** | *N-(2,4-difluorophenyl)-4-(dimethylamino)pyridine-3-carboxamide* | *C14H13F2N3O* | ** | *277.275* |
| ***S1*** | *methyl 1-(2,4-difluorophenyl)-5-(dimethoxymethyl)pyrazole-4-carboxylate* | *C14H14F2N2O4* | ** | *312.273* |

***Table S1. The pharmacophore-based screening identified fourteen virtual hits.*** *The four highlighted compounds (B2, C2, K4, and K5) demonstrated evident senolytic activity in an in vitro assay.*

**
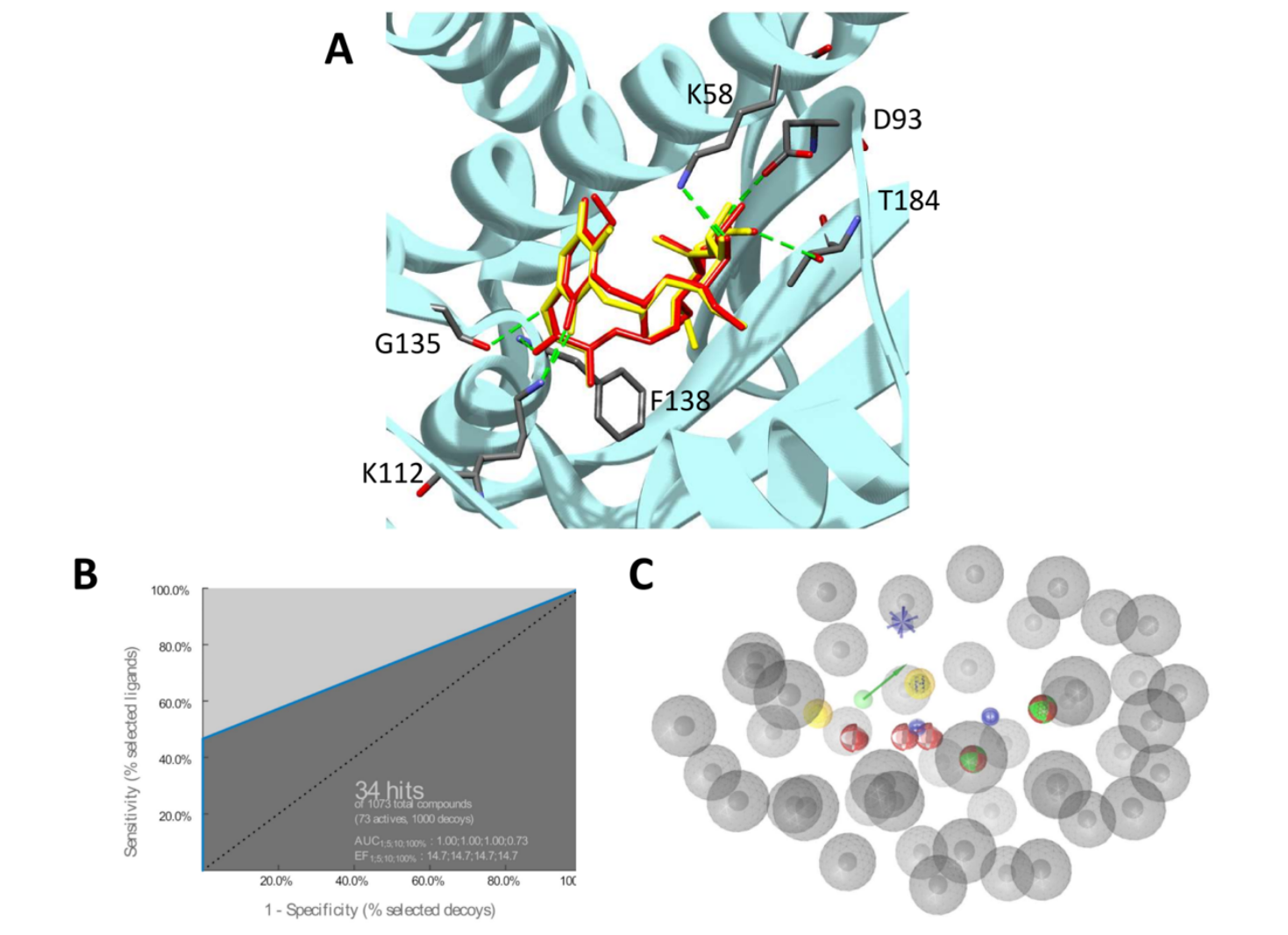
**

***Figure S1. Validation of Docking Accuracy, Screening Performance, and Pharmacophore Model Generation.*** ***(A)*** *Comparison between the predicted pose of Geldanamycin (red) and the co-crystallized ligand (yellow) pose.* ***(B) The*** *Receiver Operating Characteristics (ROC) curve was obtained by screening 73 molecules with known activities and 1000 decoys.* ***(C)*** *Pharmacophore model generated by LigandScout. The interactions were visualized in LigandScout with the following color code: HBA (red sphere), HBD (green sphere), H (yellow sphere), positive-ionizable (violet), and XVOLs (grey sphere).*

**
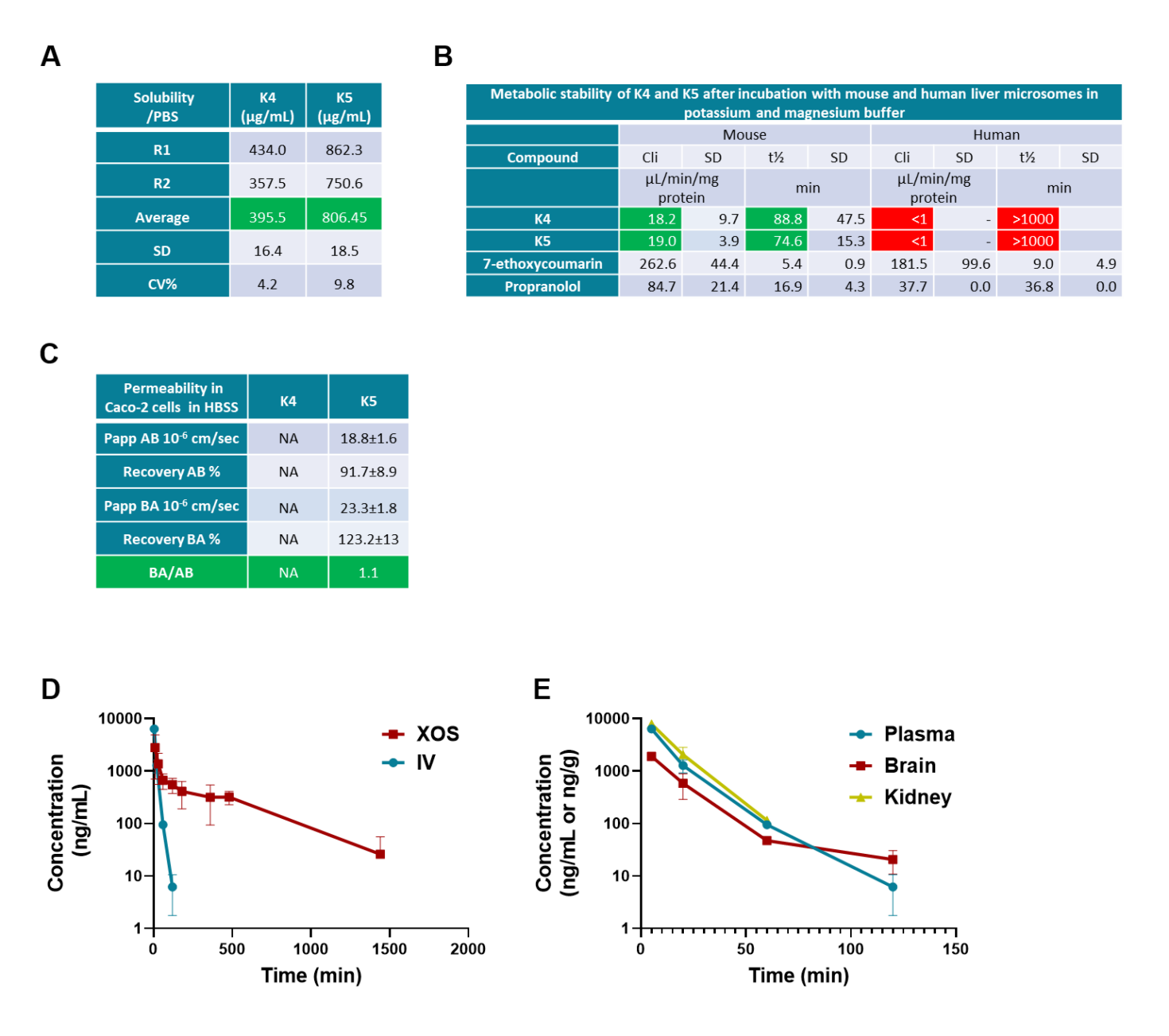
**

***Figure S2. K4 and K5 thermodynamic solubility, metabolic stability and permeability, mouse in vivo pharmacokinetics, oral bioavailability, and tissue distribution. (A-C)*** *The tables show the evaluation of K4 and K5 thermodynamic solubility in PBS at pH 7.4* ***(A)****,* *K4 and K5 metabolic stability after incubation with mouse and human liver microsomes in potassium and magnesium buffer* ***(B)****,* *and K5 permeability in Caco-2 cells in HBSS at pH 7.4* ***(C)****.* ***(D)*** *Determination of the mouse plasma exposure to K5 after oral (XOS; 30 mg/kg) and intravenous (IV; 5 mg/kg) administration.* ***(E)*** *Determination of K5 distribution in plasma, brain, and kidney after IV administration at 5 mg/kg. The graphs show the concentration profiles in a semi-logarithmic scale, n = 3; data are presented as mean ± SEM.*

***
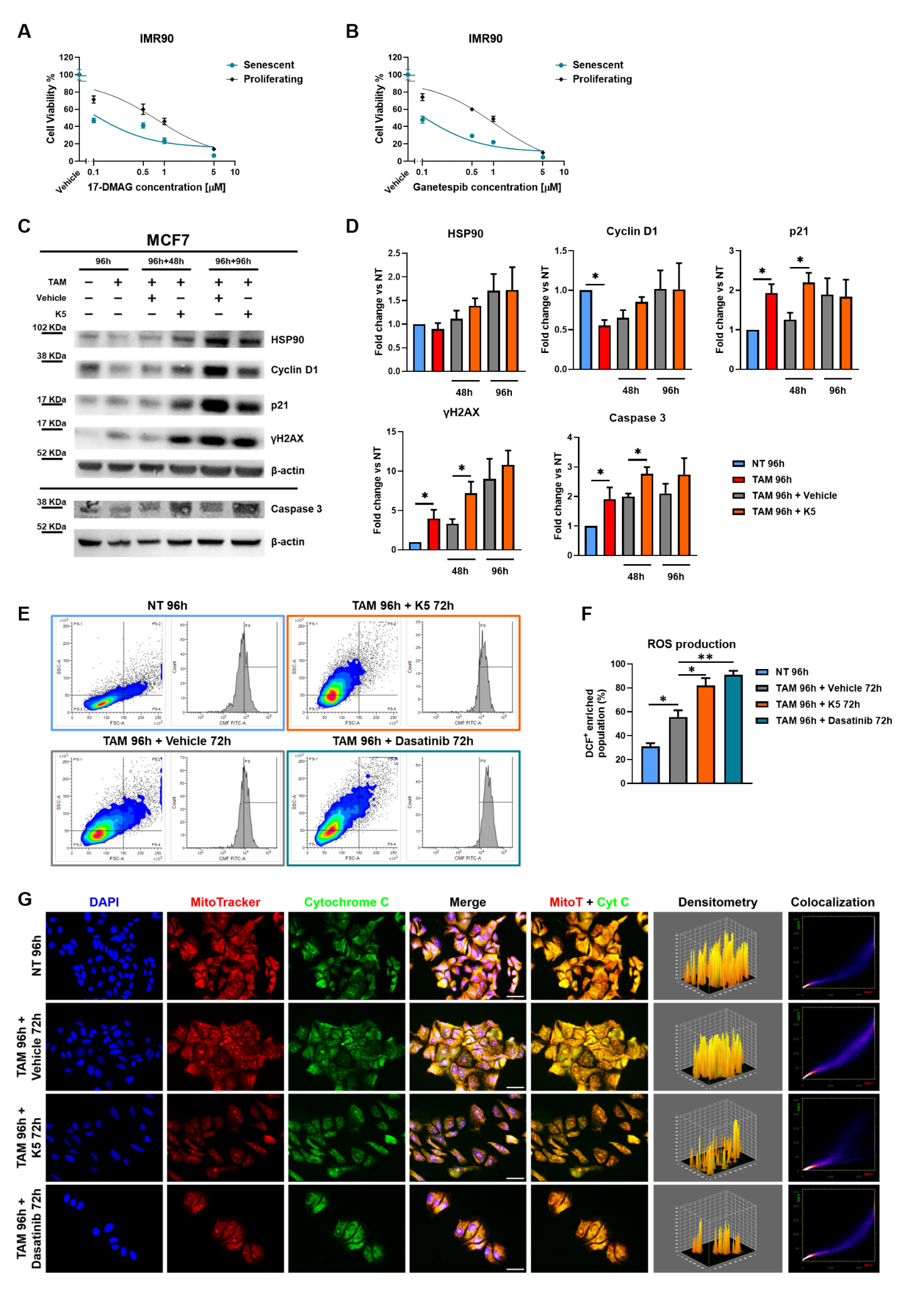
***

***Figure S3. K5 senolytic effect validation.*** ***(A-B)*** *Dose-response curves on proliferating (PD14, black lines) and senescent (PD21, blue lines) IMR90 cells treated with compound 17-DMAG and Ganetespib at increasing concentrations (0.1, 0.5, 1, and 5 μM) for 48 h. Vehicle-treated cells (DMSO) were used as solvent control. All cells were counted using Trypan blue reagent; data are presented as mean ± SEM, n = 3.* ***(C-D)*** *Effect of the senolytic drug K5 on MCF7 protein expression after Tamoxifen (TAM) treatment. Representative western blot for p21, γH2AX, HSP90, Cyclin D1, and Caspase 3, and densitometry analysis in MCF7 cells after EtOH 75% (NT; blue) or Tam (10μM; red) + K5 (10μM; orange) or Vehicle (DMSO, grey) treatment. β-actin was used as a loading control. Data plotted as fold change vs NT are represented as mean ± SEM of 3-5 independent experiments. A molecular weight marker is indicated. Statistical significance was determined using a parametric paired two-tailed Student's t-test. *p < 0.05; **p < 0.01; ***p < 0.001.* ***(E)*** *Representative contour plots and histograms visually depicting the modulation of Reactive Oxygen Species (ROS) levels in MCF7 cells after 96 h-treatment with Tamoxifen 10 µM and solvent control (NT, EtOH 75%, light blue box) and 72 h of treatment with K5 10 µM (orange box), Dasatinib 1 µM as positive control (dark blue box), and Vehicle as solvent control (DMSO, grey box), analyzed by cytofluorimetry (FACS Melody, BD).* Images were acquired with the FACS Chorus software. ***(F)*** *The graph shows the percentage of the DCF (6-chloromethyl-2′,7′-dichlorodihydrofluorescein) positive enriched population (P6 gate on the right of each histogram), analyzed by FACS, indicating the ROS modulation in MCF7 cells after 96 h of treatment with Tamoxifen 10 µM and solvent control (NT, EtOH 75%, light blue bar) and 72 h of treatment with K5 10 µM (orange bar), Dasatinib 1 µM as a positive control (dark blue bar), and Vehicle as solvent control (DMSO, grey bar). 1way ANOVA and Tukey post-hoc test analyzed data, n = 4; mean ± SEM; *p < 0,05; **p < 0,05.* ***(G)*** *Immunofluorescence representative images showing the Cytochrome C localization (green signal) in senescent MCF7 cells after* *96 h treatment with Tamoxifen 10 µM and solvent control (NT, EtOH 75%), and 72 h treatment with K5 10 µM, Dasatinib 1 µM as positive control, and Vehicle as solvent control (DMSO). DAPI (blue signal) and MitoTracker orange (red signal) were used to visualize nuclei and mitochondria, respectively. Images were acquired at 40X magnification, scale bar = 50 µm. The densitometry and the colocalization scatter plots were analyzed by ImageJ using the 3D and CoFinder plugins, showing a Pearson's Rr (PRr) = 0.956 and Overlap R (OR) = 0.963 for non-treated cells NT, PRr= 0.961 and OR = 0.978 for cells treated with TAM + Vehicle, PRr= 0.810 and OR = 0.896 after TAM + K5 treatment, and PRr = 0.928 and OR = 0.956 for TAM + Dasatinib treated cells.*


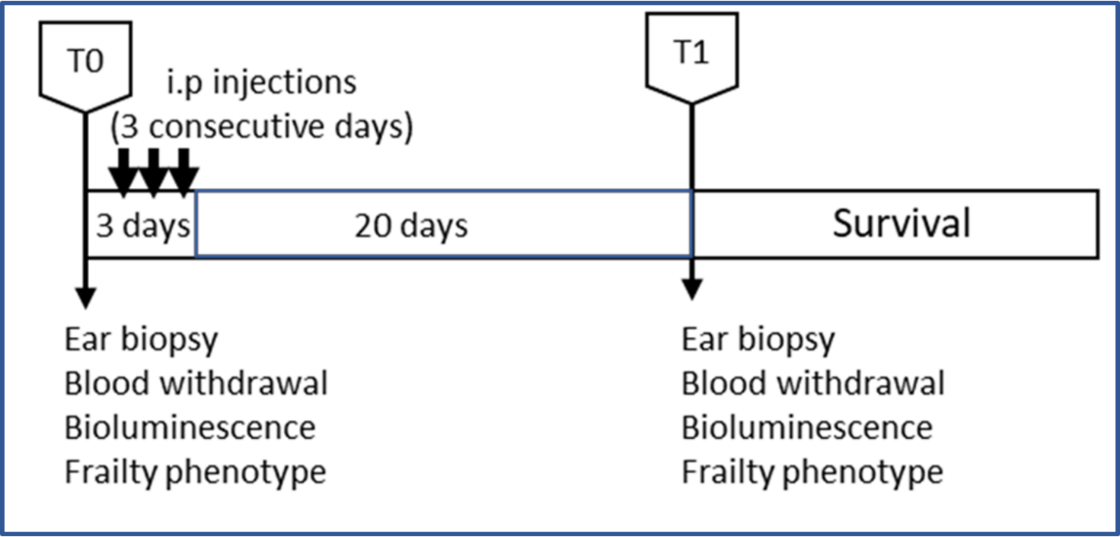


***Figure S4. A scheme of the pilot longitudinal study was performed on p16-3MR mice treated with K5.*** *The experimental population consisted of 9 mice aged 26 months (4M, 5F) treated with K5.*


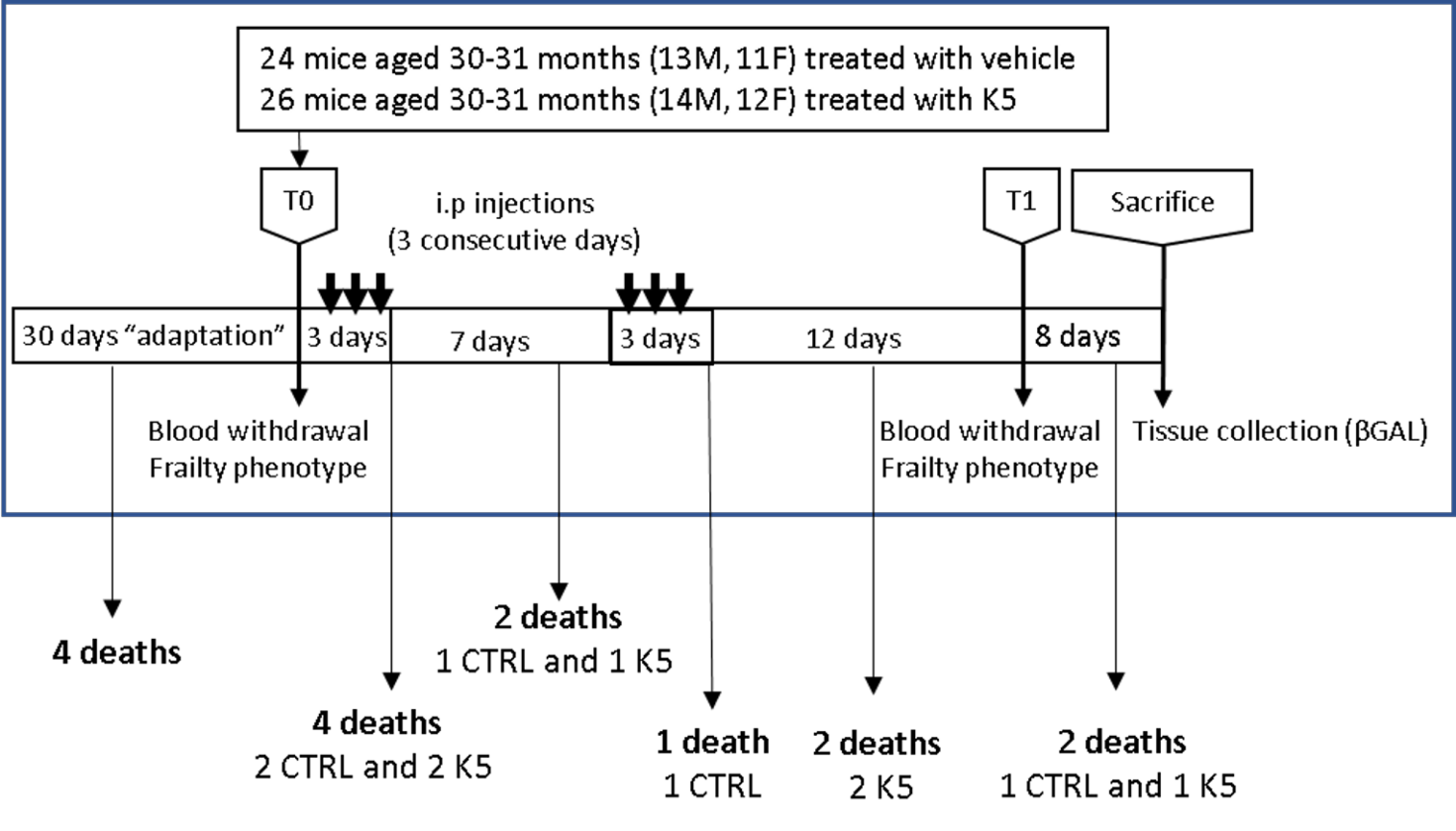


***Figure S5. The study scheme was performed in very old C57BL/6J mice treated with K5.*** *The experimental population consisted of 50 mice aged 30-31 months (27M, 23F). Deaths occurred usually because of age-related pathologies without differences between treated mice and controls.*


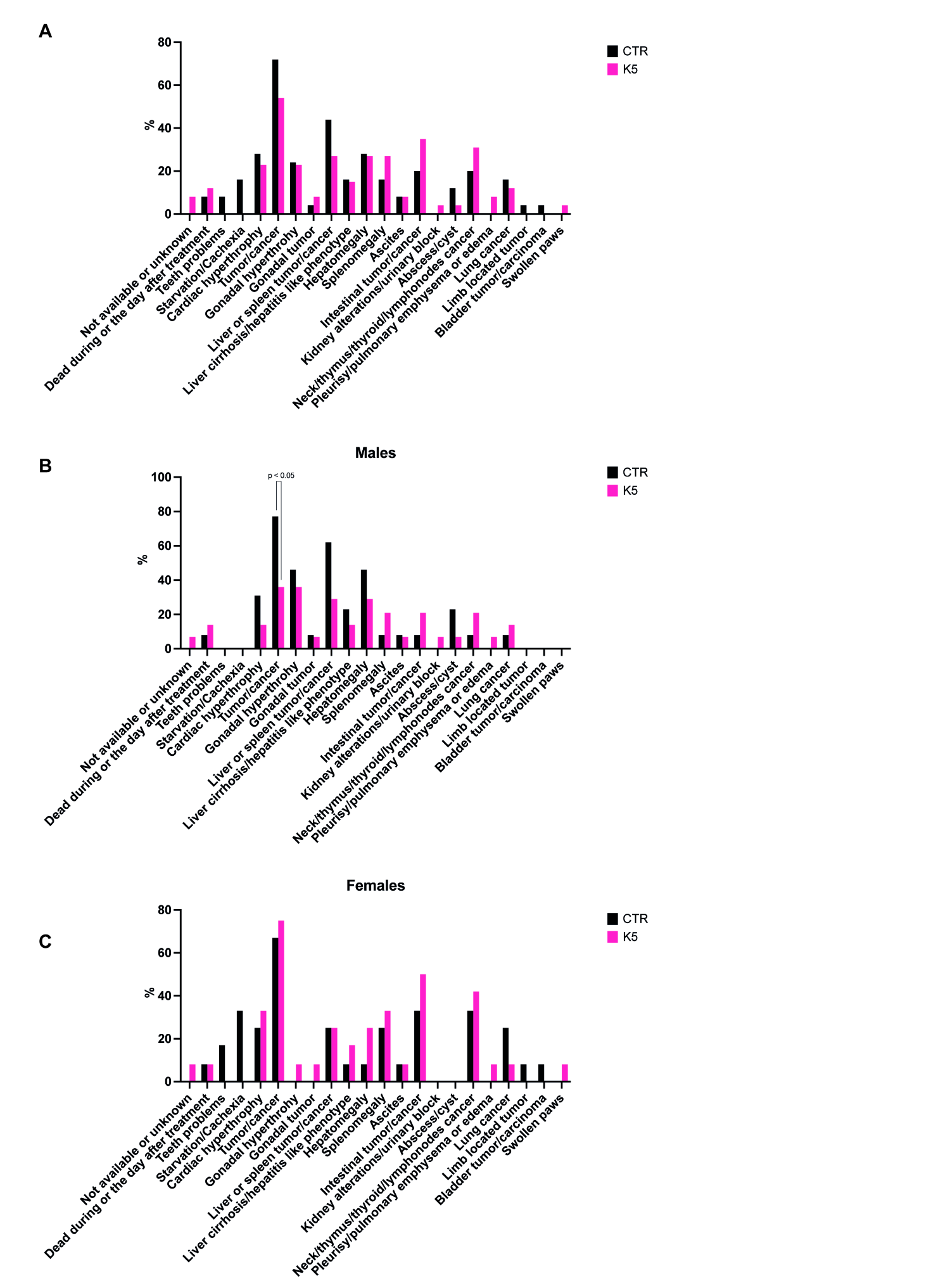


***Figure S6. Pathologies detected in geriatric mice by gross necroscopy during the explants.*** *The data are presented as three bar graphs showing pathologies detected during necroscopy: Panel* ***A*** *shows the total population (both males and females), Panel* ***B*** *shows males, and Panel* ***C*** *shows females. In males (Panel B), there appears to be a notable decrease in tumor incidence in the K5-treated group (pink bars) compared to the control group (CTR, black bars).*

## **Extended Materials and methods**

**Structure-based virtual screening.** An initial structure-based virtual screening was carried out to generate a library of compounds with the appropriate structural complementarity of the Hsp90 ATP binding site. The structure of HSP90 in complex with Geldanamycin (PDB code: 1YET) (*Stebbins et al.* 1997) was prepared using the "Protein Preparation Wizard" tool of the Schrödinger suite and used as receptor structure in docking simulations with the program Glide (Schrödinger Release 2021-4: Glide, Schrödinger, LLC, New York, NY, 2021). The NCI Diversity Set III of 2243 compounds and the Maybridge HitFinder database (14400 compounds) were merged, and the virtual library was filtered for druglike properties (Lipinski *et al*. 2001). PAINS (Pan Assay Interference Compounds) were removed by computational filtering (Baell & Nissink 2018), and the remaining compounds were prepared by Schrödinger's LigPrep tool of Maestro to apply the OPLS5 force field (Banks *et al.* 2005) to optimize the structures and to add hydrogen atoms. Epik, implemented within, was used to assign likely protonation states at pH 7.4 and tautomers to each molecule. The final minimized set of 24311 structures was submitted to ligand-flexible docking into the active site of 1YET. A ligand-flexible docking was performed at two different levels of precision, standard (SP) and the more accurate extra precision (XP) modes using default parameters. The top 50% from XP calculations (12156 molecules) were rescored based on binding energy using the Prime/MM-GBSA (Schrödinger, LLC) method (Li *et al.* 2011) and selected for further analysis. Molecular docking was validated by performing a redocking experiment and computing the root mean square deviation (RMSD) that resulted from superimposing the redocked ligand structure and the crystallographic one.

**Pharmacophore modeling.** The generated 3D virtual library was screened against a pharmacophore hypothesis based on known Hsp90 inhibitors, thus combining a structure-based approach with a ligand-based one (De Donato *et al.* 2018) LigandScout 4.1 (Inte: Ligand) (Wolber *et al.* 2006) was used for 3D pharmacophore generation, refinement, and screening *via* a ligand-based strategy. The training set contained 13 compounds with inhibitory activity against Hsp90, and the test set contained 73 active compounds and 1000 decoy molecules generated by the DUD-E web service (Mysinger *et al.* 2012). Active compounds were retrieved from the ChEMBL database (Gaulton *et al.* 2012) using an activity threshold (IC_50_) of 5 nM and 30 nm for the training and test sets. Excluded volumes representing the sterically occupied regions by the receptor were considered to increase the selectivity of the model. All LigandScout parameters were kept by default, and the best fitting model was selected and applied to the 3D virtual library of 12156 compounds representing the Glide solutions.

**Cell culture and treatment.** IMR90 cells (*Homo sapiens*, fetal lung, fibroblast, Caucasian) and WI38 cells (*Homo sapiens*, fetal lung, fibroblast, Caucasian) were purchased from ECACC-Merck (Merck, Darmstadt, Germany) and cultured in MEM medium (Euroclone, Milano, Italy) supplemented with 1% L-Glutamine (Euroclone, Milano, Italy), 1% Penicillin-Streptomycin (Euroclone, Milano, Italy), and 10% Fetal Bovine Serum (FBS, Euroclone, Milano, Italy), at 37°C, 5% CO_2_. IMR90 cells with a population doubling (PD) from PD15 to PD23 were used; cells were considered incrementally senescent from passage PD19 to passage PD23. WI38 cells were used from PD26 to PD53. Cells were considered incrementally senescent from passage PD49 to passage PD53. For the compounds *in vitro* screening, IMR90 cells with passages from PD15 to PD23 were tested at increasing concentrations of 0.5 μM, 1 μM, 10 μM, and 25 μM over 24 and 48 h to evaluate the compounds' efficacy and cytotoxicity compared to the Vehicle (DMSO ≤ 0.1%). Cells were counted manually, in triplicate, using Trypan blue reagent (Euroclone) and disposable Bürker chambers. The reference compounds 17-DMAG (Selleckchem) and Ganetespib (Selleckchem) were also used as controls of the experiments (Fuhrmann-Stroissnigg *et al.* 2017). K5 dose-response proliferation curves were performed with compound K5 (Molport, SIA, Riga, Latvia) in concentrations 0.1, 0.5, 1, 5, 10, 50, 100, and 200 μM for 48 h. Cells were counted using a Trypan blue reagent and EVAplus cell counter. The EC_50_s were calculated by analyzing the data using GraphPad Prism 10 software.

Human bone marrow (BM)-derived mesenchymal stromal cells (MSCs) were purchased from Lonza (Allendale, NJ, USA) and cultured in α-MEM (Euroclone, Milano, Italy), with 10% fetal bovine serum (FBS, Lonza, Stein, Switzerland), 100 U/mL penicillin, 100 mg/mL streptomycin and 2 mM L-glutamine. Cells were seeded at a density of 5000/cm^2^ and maintained at 37°C in a humidified atmosphere with 5% CO_2_. The culture medium was changed at 48 h intervals, and cells were trypsinized when almost 80% were confluent using 0.25% trypsin-EDTA (Euroclone, Milano, Italy).

Human umbilical vein endothelial cells (HUVECs) are primary cells obtained from a pool of donors purchased from Clonetics (Lonza, Stein, Switzerland). HUVECs were cultured in endothelial basal medium (EBM-2, CC-3156, Lonza) supplemented with SingleQuot Bullet Kit (CC-4176, Lonza) containing 0.1% human recombinant epidermal growth factor (rh-EGF), 0.04% hydrocortisone, 0.1% vascular endothelial growth factor (VEGF), 0.4% human recombinant fibroblast growth factor (rh-FGF-B), 0.1% insulin-like growth factor-1 with the substitution of arginine for glutamic acid at position 3 (R3-IGF-1), 0.1% ascorbic acid, 0.1% heparin, 0.1% gentamicin and amphotericin-B (GA-1000), and 2% fetal bovine serum (FBS). The cells were seeded at a density of 5000/cm^2^ in T75 flasks (Corning Costar, Sigma Aldrich, St. Louis, MO, USA).

Murine ear fibroblasts (MearFs) were obtained from C57BL/6J mice maintained in the INRCA "Specific Pathogen Free" (SPF) animal facility. The ear biopsies (2 mm radius) were obtained with a dedicated puncher, and cells recovered as previously described (Malavolta *et al.* 2022). Cells were seeded at a density of 5000/cm^2^ in T75 flasks (Corning Costar, Sigma Aldrich, St. Louis, MO, USA) and maintained at 37°C in a 95% humidified incubator with 5% CO_2_. Culture was carried out in RPMI (Euroclone, Milano, Italy) supplemented with 10% Fetal Calf Serum (FCS, Sigma-Aldrich, St. Louis, MO, USA), 2mM L-Glutamine, 100 U/mL penicillin and 100 mg/mL streptomycin (all from Sigma- Aldrich, St. Louis, MO, USA). At approximately 80% confluence, the cells were detached from the plate by incubation with trypsin-EDTA (Sigma-Aldrich, St. Louis, MO, USA) for 5 min at 37 °C and re-plated. For Doxorubicin-induced senescence, cells were seeded in a 96-well plate at a 5000 cells/cm2 density. MSC cells were treated with 100 nM Doxorubicin (DOX) for 24 h and then maintained with medium for 4 days; HUVECs were treated with 50 nM DOX for 24 h and then maintained with medium for 4 days. MearFs were induced to senescence by continuous exposure (5 days) to 75 nM DOX.

After senescence induction, MSCs, HUVECs, and MearFs were incubated with 0.5, 1, 5, 10, 50, 100 or 200 μM of K5 for 48 h.

MCF7 cell line (ATCC; HTB-22, *Homo sapiens*, breast cancer) was purchased from ATCC; cells were grown in MEM medium (Corning, New York, USA, #15-010-CVR) supplemented with 10% FBS (Gibco, Invitrogen, Carlsbad, CA, USA, #10270106), 1% glutamine (Corning #25005-CI), 1% penicillin and streptomycin (Corning #30002-CI). Cells were incubated at 37 °C with 5% CO_2_. Senescent status induction: MCF7 were plated 24 h before the treatment with 4-Hydroxytamoxifen (TAM) 10 μM to induce the senescence described by (Lee *et al.* 2014). Control cultures were treated with equal volumes of 75% ethanol as the vehicle. The treatment with TAM lasts 96 h, after which the cells have been washed and treated with the senolytic drugs K5 10 µM for another 96 h. The treatment with the senolytic compounds has been renewed at 48 h. Cell mortality and vitality curves after treatment with K5 (10 μM, orange) or Vehicle (DMSO, grey) were monitored using the Incucyte live-cell Analysis system (Sartorius, Goettingen, Germany). The estimated Senolytic index (SI) was calculated as the ratio between the EC_50_ in proliferating cells and the EC_50_ in senescent cells, extrapolated using GraphPad Prism 10 software.

**Cell viability assay.** Cell viability has been determined with MTT (3-(4,5-dimethylthiazol-2-yl)-2, 5-diphenyltetrazolium bromide) assay. MTT (5 mg/mL) solution was added to each well (10 µL/100 µL medium) and incubated for 4h. Insoluble formazan salts produced were solubilized with DMSO incubation, and then absorbance was measured at 540 nm using a microplate reader (NB-12-0035 Microplate Reader, NeoBiotech Co., Seoul, Republic of Korea). Data are expressed as a percentage of viability compared with untreated cells (the control group has been considered 100% of viability).

**β-galactosidase colorimetric assay.** For cell cultures: Previously treated cells were fixed with the fixative solution for 10 min at RT according to the protocols established by the supplier (Senescence β-Galactosidase Staining Kit, #9860S, Cell Signaling, Danvers, MA, USA) Cells were then incubated with the substrate, X-gal, in a solution at pH 6, at 37°C, overnight (16 h) (Itahana *et al.* 2007). The images were acquired using an optical microscope (Leica, Wetzlar, Germany) with 5 and 10X magnification or the phase microscopy EVOS XL Core at 10X magnification. The images were analyzed using the ImageJ software, quantifying the colorimetric signal emitted by each cell. The mean signal intensity was then normalized for the total number of cells.

For aged tissues: 10 μm-thick tissue sections were prepared from nine organs (liver, heart, lung, kidney, quadriceps, gastrocnemius, brain cortex, cerebellum, and hippocampus) and ear biopsies with a cryostat from snap-frozen tissues and mounted on SuperFrost Plus slides (VWR, Radnor, PA, USA). SA-β-gal staining was performed according to the manufacturer's instructions of the Sigma-Aldrich-QIA117 staining kit (Sigma-Aldrich, St. Louis, MO, USA). Nuclei were counterstained with Nuclear Fast Red (NFR) (Sigma-Aldrich, St. Louis, MO, USA), and images (from 0.77 µM × 0.77 µM sections) were acquired using a Zeiss AxioCam HRc mounted on a Leitz Laborlux S light microscope. The percentage of senescent cells was determined as the mean of 3–4 images from each tissue section by counting the total and SA-β-gal-positive cells with the positive cell detection tool available in the open-source software for digital image analysis, QuPath v. 0.3.2 (Bankhead *et al.* 2017).

**HSP90 inhibition activity assay.** HSP90-directed inhibitory activity was assessed employing fluorescence polarization measuring competitive binding of fluorescein isothiocyanate-labeled Geldanamycin (FITC-Geldanamycin) to recombinant HSP90α as a function of test compound concentration using the commercially available HSP90 N-Terminal Domain Assay Kit ( #50293, BPS Bioscience, CA, USA) according to the manufacturer's instructions. Briefly, the compounds were diluted at different concentrations between 1 pM to 10 μM and incubated for 2 h with the enzyme and substrate at RT while stirring. The fluorescence intensity was measured with a multiplate reader (Victor Nivo-Perkin Elmer) set at 485/530 nm. Data are the percentage of residual enzyme activity relative to the solvent (DMSO). The IC_50_s were calculated by analyzing the data using GraphPad Prism 10 software.

**Cellular Thermal Shift Assay (CETSA).** CETSA was performed according to standard protocols (Jafari *et al.* 2014). Briefly, the cellular extract was lysed in RIPA buffer (10 mM Tris-HCl pH 7.4, 250 mM NaCl, 1% NP40/IGEPAL, 1% DOC, 0.01% SDS, 0.01% Glycerol, supplemented with protease/phosphatase inhibitor, PI/PhI mix and 2 mM DTT) and protein concentration was determined by a BCA kit (Pierce, Life Technologies, TFS, Waltham, MA, USA). Samples were homogenized by 3 cycles of freeze-thawing using liquid nitrogen and centrifuged at 13000 rpm at 4 °C for 30 min to separate the soluble fraction from the debris. Next, the lysates were divided into aliquots incubated with the compound of interest (DMSO, K4, K5, Caffeine, and Ganetespib, at 500 μM for 1 h at RT. Each aliquot was further divided into smaller volumes (20 μL) and heated individually at increasing temperatures (0°C, 46°C, 70°C) for 3 min, followed by 3 min cooling at RT, and finally centrifuged at 13000 rpm, for 30 min, at 4 °C. All supernatants were then separated from precipitates and analyzed by capillary electrophoresis.

**Capillary electrophoresis-Western blot automation (WES).** Following the supplier's recommendations, samples were loaded into the proper cartridge (12- 230 kDa Wes/Jess Separation Modules, ProteinSimple, Biotechne, Minneapolis, MN, USA). HSP90α (1:50, MsMAb, Abcam), HSP90β (1:50, RbMAb, Abcam), GRP94 (1:50, RbMAb, Abcam), TRAP1 (1:50, MsMAb, Abcam), α-Tubulin (1:500, MsMAb, Abcam), and β-Actin (1:500, RbMAb, Abcam) were used as primary antibodies. Secondary antibodies were Anti-Mouse Secondary HRP and Anti-Rabbit Secondary HRP (ProteinSimple, Biotechne, Minneapolis, MN, USA). For representative blots, images at different contrasts were exported to represent better the area measurements automatically calculated by the "Compass for SW" program.

**Western Blot.** WB was performed according to standard procedures. Total protein extract was performed as in (Aiello *et al.* 2016). Western blot assay used 10 µg of protein extract and proteins solved by SDS-PAGE. Protein signals were revealed with ECL Prime (Amersham, GE Healthcare, Boston, MA, USA) and detected by UVITEC (Eppendorf S.r.l., Hamburg, Germany). Densitometric analysis was performed with NIH Image J 1.8 software (National Institutes of Health, Bethesda, MD, USA), and specific values were normalized to loading control (β-Actin).

**Immunofluorescence staining**. Treated/untreated IMR90 and MCF7 cells were cultured in LabTek II chamber slides (Nunc, Biosigma). At the final time point, cells were stained with 500 nM MitoTracker Orange (Thermo Fisher Scientific) for 30 min at 37°C and then fixed in 4% PFA (Merck) for 10 min, RT, and permeabilized with 0.1% Triton solution (Merck) for 30 min at RT. Samples were blocked in 5% BSA for 90 min, incubated overnight at 4°C with primary antibody against Cytochrome C (1:50, RbMAb, Abcam), and 1h at RT with secondary antibody Alexa Fluor 488 goat anti-Rb (1:200, Thermo Fisher Scientific). Nuclei were stained with DAPI (Cell Signaling) for 10 min at RT, and ProLong Glass Antifade Mountant (Thermo Fisher Scientific) was used to seal coverslips. Images were acquired using an Olympus IX83 research inverted microscope at 40X magnifications.

**Immunohistochemistry**. Treated/untreated MCF7 cells cultured on coverslips were fixed in 4% PFA (Merck) for 15 min at 4˚C, followed by permeabilization with 0.2% Triton X‑100 for 4 min. Coverslips were placed on the slides, which were treated with 3% hydrogen peroxide in methanol to block endogenous peroxidase and incubated in a humidified chamber for 60 min at RT, using the primary antibody Ki67 (1:50, RbPAb, Abcam). The slides were washed with PBS and incubated for 30 min at RT with the polyvalent secondary antibody from the UltraVision Quanto Detection System horseradish peroxidase DAB kit (Thermo Fisher Scientific) and streptavidin‑peroxidase (Thermo Fisher Scientific) for 10 min using a DAB chromogenic kit (Agilent Technologies). Images were obtained using the Nikon ECLIPSE Ei R phase microscopy at 40X magnification.

**Annexin V staining.** MCF7 were plated 24 h before the treatment with 4-Hydroxytamoxifen (TAM) 10 μM for 96 h to induce the senescence described by (Lee *et al.* 2014). Control cultures were treated with equal volumes of 75% ethanol as the vehicle. Then, cells were washed and treated with the senolytic drug K5 10 µM and DMSO as solvent control for another 96 h and with the Incucyte Annexin V NIR Dye (1:200, Sartorius). Cells were monitored using the Incucyte live-cell Analysis system (Sartorius). The images were acquired using the phase microscopy EVOS XL Core at 10X magnification and analyzed using the ImageJ software. The mean signal intensity was then normalized for the total number of cells.

**Fluorescence Activated Cell Sorting (FACS).** Reactive Oxygen Species (ROS) production was evaluated by adding the compound 6-chloromethyl-2′,7′-dichlorodihydrofluorescein diacetate, acetyl ester (CM-H2DCFDA, Thermo Fisher Scientific) in PBS 1X to the cells, according to t manufacturer's instructions. Specifically, treated/untreated MCF7 cells were treated for 30 min at 37°C with CM-H2DCFDA. The cell CM-H2DCFDA is transformed in a less cell-permeable probe by cellular esterases, which reacts with ROS to yield the highly fluorescent form DCF. Cells were washed twice with PBS1X, harvested, and centrifuged at 1500 rpm for 5 min at 4°C. Finally, the pellet was resuspended in 300 μL of 1% BSA and transferred in FACS tubes. Samples were analyzed by flow cytometry in the fluorescein isothiocyanate (FITC) channels using a FACS Melody (BD Biosciences). The BD FACS Chorus Software determined the percentage of DCF-positive cells.

**Thermodynamic solubility.** The shake flask method measured the thermodynamic solubility of K4 and K5 in PBS buffers at pH 7.4. A saturated solution of the compounds was prepared by incubating the 2 compounds in PBS buffer for 24 h at 37°C under agitation. At the end of the incubation, samples were centrifuged, two aliquots were diluted in acetonitrile (CAN), and each sample was then injected into LC-MS/MS (ESI interface) and quantified on a calibration curve prepared by sequential dilution in ACN. Samples were analyzed on UPLC Acquity (Waters) interfaced with API 3200 (AB Sciex) Triple Quadrupole Mass spectrometer. For K4, a Synergy Polar C18 column was used (50x2, 1mm 5µm, Phenomenex, Temp 35°C, Inj volume 10 µL, mobile phase A: water +0.1% FA, and phase B: ACN 0.1% FA, gradient: 5%-98% B, flow: 0.3 mL/min). For K5, a Gemini NX C18 column was used (50x2, 1mm 5µm, Phenomenex, Temp 35°C, Inj volume 10 µL, mobile phase A: 5mM ammonium formate +0.1% FA, and phase B: MeOH +5%, gradient: 5%-98% B, flow: 0.3 mL/min).

**Phase I stability in human and mouse liver microsomes.** The metabolic stability of K4 and K5 was evaluated after incubation with mouse and human liver microsomes. Briefly, the 2 compounds were dissolved in DMSO and pre-incubated, at the final concentration of 1µM, for 10 min at 37°C in potassium phosphate buffer 50 mM, pH 7.4, 3 mM MgCl_2_, with mouse and human liver microsomes (Sigma) at the final concentration of 0.5 mg/mL. After the pre-incubation period, reactions were started by adding the cofactors mixture (NADP, Glc6P, Glc6P-DH in 2% Sodium bicarbonate); samples were taken at time 0, 10, 20, 30, and 60 min, and added to ACN with Verapamil 0.1 µM as Internal Standard (IS) to stop the reaction. After centrifugation, the supernatants were analyzed using LC-MS/MS. A control sample without cofactors was added to check the stability of test compounds in the matrix after 60 min. 7-Ethoxycoumarin (7-EC) and propranolol were added as positive reference standards. Samples were analyzed on Acquity UPLC (Waters) coupled with an API 3200 Triple Quadrupole ABSciex. For K4, a Synergy Polar C18 column was used (50x2, 1mm 5µm, Phenomenex, RT, Inj volume 10 µL), and for K5, a Gemini NX C18 column was used (50x2, 1mm 5µm, Phenomenex, RT, Inj volume 10 µL). Mobile phase A: deionized water + +0.1% HCOOH, phase B: ACN + +0.1% HCOOH (gradient: 5%-98% B, flow 0.3 mL/min). The percent of the area of the test compound remaining at the various incubation times was calculated based on the area of the compound at time 0 min. The rate constant, k (min^-1^), derived for the exponential decay equation (peak area/IS *vs* time), was used to calculate the rate of intrinsic clearance (Cli) of the compounds.

**Permeability in Caco-2 cells.** To evaluate the bidirectional permeability of the 2 compounds, Caco-2 cells (ECACC, ReadyCells, Barcellona, Spain), considered a reference cellular line for *in vitro* preclinical evaluation of oral dosage products, were used. These cells are cultured in monolayers, which become differentiated polarized epithelial cells after 21 days, expressing tight junctions, microvilli, intracellular metabolic enzymes, and transmembrane transporters such as BCRP, MDR1, and MRP2 (an artificial model equivalent to the intestinal epithelium). Cells are then plated in 24-Insert HTS initial TEER >1000Ω (Millicell-ERS Millipore). HBSS buffer was placed in the apical compartments and the basolateral compartments. Compound solutions were spiked in the apical compartments for apical to basolateral transport (A→B) and basolateral compartments for basolateral to apical transport (B→A) to reach the final concentration of 5µM. Samples at T=0 from the donor compartments were diluted with an Internal standard (IS) solution (Verapamil 10 ng/mL in ACN). After 2 hours of incubation at 37°C and 5% CO2, the apical was diluted with IS solutions on the basolateral side. At the end of the experiment, the integrity of the cellular junction was measured by incubating the Lucifer yellow (LY) at the apical side (at the concentration of 0.02 mg/mL in MEM medium) and with MEM medium in the basolateral compartment for 1 hour at 37°C. At the end of incubation, fluorescence was read on a fluorimeter at λ 430-538nm. Samples were analyzed on a UPLC Acquity (Waters) coupled with an API 3200 Triple Quadrupole AB Sciex. For K4, a Synergy Polar C18 column was used (50x2, 1mm 5µm, Phenomenex, RT, Inj volume 10 µL), and for K5, a Gemini NX C18 column was used (50x2, 1mm 5µm, Phenomenex, RT, Inj volume 10 µL). Mobile phase A: deionized water + +0.1% HCOOH, phase B: ACN + +0.1% HCOOH (gradient: 5%-98% B, flow 0.3 mL/min). The apparent permeability (Papp), the efflux ratio (ER), and the Recovery (R) were calculated to allow for estimation of metabolism and non-specific binding. To ensure suitable functionality of the monolayers, the following controls were applied in the study: the permeability of LY, known as low permeability compound, indicates the proper integrity of the monolayer; LY values are accepted if below 0.7% permeability; the efflux ratio >2 suggest P-gp mediated or other transporters efflux phenomena, recovery should be > 75%. Data are expressed as mean of n=2 (± SD).

**Determination of the mouse plasma exposure of K5 after oral (XOS) and intravenous (IV) administration. Determination of K5 distribution in brain and kidney after IV administration.** Weighed tissues were homogenized with a Precellys Evolution Homogenizer 1g/5mL of 20 mM ammonium formate buffer pH 6.5 using 3 cycles 25 sec at a speed of 7600 rpm. Plasma or tissue was added to ACN containing Verapamil as Internal standard (0.1 µM) in a 96 healthy plate. Samples were shaken for 10 min at 300 rpm at RT and centrifuged for 15 min at 10°C at 4700 rpm. Working solutions (WS) for the calibration curves and QC samples were prepared by diluting ACN in the range 10-20000ng/mL range. Verapamil was diluted in ACN to reach the final concentration of 0.1 µM (WS_IS). Samples were analyzed on an Acquity UPLC (Waters) coupled with an API 3200 Triple Quadrupole ABSciex. Non-compartmental analysis was applied, and the following pharmacokinetic (PK) parameters were evaluated for each subject or group of subjects: maximum plasma concentration (Cmax), plasma concentration at last timepoint (Clast), time of maximum plasma concentration (Tmax), AUC from time zero to the time of the last quantifiable plasma concentration (AUC0-last), AUC from time zero extrapolated to infinity (AUCinf), mean residence time (MRT), half-life (T½). AUCs were calculated using a linear trapezoidal rule, and a uniform weight was performed as the first general approach. Graphical concentration-time curves are produced after Log transformation (PK Solver 2.0, Excel 2007 Microsoft add-in). The ke was estimated from the terminal part of the log-concentration time plot, which included at least three data points excluding the Cmax. If the terminal phase regression based on R_2_ was less than 0.85, then the T½ and the AUC0-inf were not reported. If the AUC 0-t/0-inf was less than 0.8, then the AUC0-inf was not reported.

***Drosophila melanogaster* maintenance and lifespan assays.** All experiments were carried out with wild-type Canton-S *Drosophila melanogaster*. Canton-S flies originated (Stock #64349) from the Bloomington *Drosophila* Stock Centre (Indiana University, Bloomington, IN, USA). Flies were reared on Nutri-Fly Bloomington Formulation food medium (Genesee Scientific, El Cajon, CA, USA) prepared using 176 g of Nutri-Fly per liter of distilled H_2_O. After cooling to 70°C, 4.8 mL of propionic acid (Merck KGaA, Darmstadt, Germany) per liter of food was added. Flies were maintained at constant temperature (25.0 ± 0.2°C) with 60% relative humidity on a 12 h:12 h 300 lux light-dark cycle generated by a 4000°K LED lamp (Fridge 1.5W, Ecoman Italia S.r.l., Frascati, Italy).

For lifespan assays, newly enclosed *Drosophila melanogaster* (1 to 2 days old) were collected and placed separately in vials (each containing 10 male and 10 female flies). Two experiments were performed. In the first experiment, 300 flies were randomly divided into three groups (CTRL, K5 10 µg/mL, and K5 100 µg/mL). Vials containing only fresh food (Nutri-Fly Bloomington Formulation food medium) were alternated 3 times a week with vials containing food supplemented with K5. The K5 was dissolved in DMSO and then diluted 1:10 in corn oil. 5 µL of corn oil (containing 0, 10 µg, or 100 µg of K5) was added to each mL of freshly prepared Nutri-Fly Bloomington Formulation food medium. In the second experiment, 900 flies were divided by sex into CTRL group and K5 10 µg/mL treated group. Until the 19th day of life, all flies were fed standard food. Starting from the 20th day of life, the flies were maintained in vials with standard food alternated (3 times a week) with vials containing food and 0 or 10 µg/mL of K5 prepared as described before. All experiments counted the number of living flies every 2–3 days. Flies that failed to respond to taps were scored dead, and those stuck to the food were censored.

***Drosophila melanogaster* gene expression analysis.** Total RNA from *Drosophila melanogaster* was isolated using a TripleXtractor reagent kit (Grisp Research Solution, Porto, Portugal) based on the phenol and guanidine isothiocyanate extraction. The flies were divided into two groups (CTRL and K5 10 µg/mL treated group). K5 treatment started from the 20^th^ day of life, as described in the second lifespan experiment. On the 27^th^ day of life, fruit flies were killed by freezing them at -20°C for 20 minutes and then transferred in a 1.5 ml Eppendorf tube (Costar, Corning, NY, USA). The tubes were immersed in liquid nitrogen for 5 minutes and crushed with a pestle (Euroclone, Milan, Italy) in 1 ml of TripleXtractor reagent. RNA was extracted from lysate following the manufacturer's instruction of TripleXtractor reagent kit, quantified with a Nanodrop (NanoDrop 1000, Thermo Fisher, Waltham, MA, USA), and stored at −80°C until use. According to the manufacturer's instructions, one microgram of total RNA was reverse-transcribed using a Prime Script RT Reagent Kit with the gDNA Eraser (Takara, Göteborg, Sweden). Quantitative real-time PCR (qRT-PCR) was conducted using a QuantStudio™ 1 Real-Time PCR System (Thermo Fisher) instrument. The amplification of *Drosophila melanogaster* *Glyceraldehyde-3-phosphate dehydrogenase 1* (*Gapdh1*; used as reference) and *Dacapo* (*Dap*), *Unpaired 2* (*Upd2)*, *Unpaired 3* (*Upd3*) was performed using Excel-Taq FAST qPCR SybrGreen (SMOBIO Technology Inc, Neuenburg am Rhein, Germany), according to the manufacturer's instructions. Primer sequences were taken from FlyPrimerBank (www.flyrnai.org/flyprimerbank). The relative quantity of mRNA specific to each of the target genes was calculated using the 2^−ΔCT^ (relative expression) method: ΔCt = ΔCt (CTRL) − ΔCt (K5 treated). The relative expression values of the genes of interest were reported as mean ± standard deviation (SD) of three independent experiments.

**Mice and experimental design.** All experiments were performed per the European Community Council Directives of 2010/63/UE. Two experiments were conducted on aged mice: Pilot study to investigate safety and potential senolytic efficacy and an Extended study to investigate functional changes, pathological phenotype, and the principal organ targeted by K5. The protocols were approved according to current Italian law (*D.Lgs.* n. 26/2014) by the Organismo Preposto al Benessere Animale (OPBA, animal care and health committee) of IRCCS INRCA and by the General Direction of Animal Health and Veterinary Drugs of the Italian Ministry of Health with the authorization no.137/2021-PR for the Pilot study and authorization n^o^—370/2022-PR for the Extended study. Additional control mice used to compare survival and phenotype data were approved according to authorization no. 392/2019-PR.

In the pilot study, nine p16-3MR mice (4 males and 5 females, aged 26 months) received intraperitoneal K5 treatment (at a dosage of 10 mg/kg), administered through three injections over three consecutive days (**Fig. S4**). To explore the safety of K5 and its potential impact on the aging process, p16-3MR mice were longitudinally studied in this pilot experiment. They were monitored once a month until natural death using a non-invasive protocol designed to assess their clinical health and physical performance across various functional domains associated with frailty. Additionally, to provide an initial overview of K5 effects on senescent cells, non-invasive and exploratory analyses (senescence markers in ear biopsies, p16 expression measured by *in vivo* bioluminescence assay, and circulating cytokine profile) were performed before the start of the treatment (T0) and after 20 days following the end of K5 administration (T1).

In the extended study, more geriatric C57BL/6J mice were used to confirm safety and investigate the main organs targeted by K5 (**Fig. S5**). For this purpose, 26 geriatric mice (14 males, 12 females, aged 31 months) and 24 geriatric mice (13 males, 11 females, and 31 months) were treated with K5 and the Vehicle. In this experiment, the animals underwent two rounds of treatment, with a 7-day interval, each consisting of three injections administered over three consecutive days. In all animals, clinical health, physical performance, and circulating cytokines and chemokines were analyzed both before and after the end of the treatment period. Subsequently, the animals were euthanized, gross necropsies were performed, and organs were collected for further analysis.

**Circulating cytokines assay.** Blood samples were collected from the right retroorbital plexus of anesthetized mice. Blood samples were deposited in serum separator gel tubes (Microvette CB300, Sarstedt) and centrifuged (1000 × g, 10 min) for serum separation. Concentrations of glucose (GLUC), creatinine (CREA), aspartate aminotransferase (AST/GOT), and alanine aminotransferase (ALT/GPT) were determined in EDTA-plasma by using an automated analyzer (Cobas Mira, Roche) according to the manufacturer's instructions. Standard controls were run before each determination. Cytokines were measured in EDTA-plasma obtained from (50-100 µl) blood using the ProcartaPlex™ Mouse Cytokine & Chemokine Panel 1, 26plex (Thermofisher) and read in the Luminex 200 instrument (Bio-Rad, Hercules, CA, USA). The plasma was collected and centrifugated at 1000× g for 15 min at 4 °C; the procedure followed was the producer's recommended. A 96-well plate was filled with 50 µL standard, control, and samples. The various reagents (microparticle cocktail, diluted biotin-antibody cocktail, and diluted streptavidin-PE) were added, alternating with incubation and washing steps.

**p21 and p53 mRNA expression in ear biopsies.** Total RNA was extracted from ear biopsies using the RNeasy kit (Qiagen, Hilden, Germany) according to the manufacturer's instructions and quantified by a NanoDrop spectrophotometer. According to the manufacturer's guidelines, cDNA synthesis from total RNA was performed using i-Script reverse transcriptase (Biorad, Hercules, CA). The resulting cDNA was subjected to real-time PCR assay to detect the expression levels of the β-actin housekeeping gene and p-21 and p-53. The primers used are the following:

β-actin: forward 5’-TTCGTTGCCGGTCCACAC-3’, reverse 5’-ACCAGCGCAGCGATATCG-3’

p-21: forward 5’-GCTGTCTTGCACTCTGGTGT-3’, reverse 5’-TCTGCGCTTGGAGTGATAGA-3’

p-53: forward 5’ TGGAAGACTCCAGTGGGAAC 3’, reverse 5’ TTTCTTCTTCTGTACGGCGG 3’

0.5 µg of cDNA was amplified in a total volume of 20 µl containing iQ SYBR GREEN SUPERMIX (Biorad, Hercules, CA) on a BioRad iQ5 optical real-time PCR (Biorad, Hercules, CA), employing a primer concentration of 150 nM (β-Actin), and 200 nM for the other genes. Assays for each transcript were carried out as duplicates. Normalization to the housekeeping gene corrected any RNA input or reverse transcription inefficiencies. Relative amounts of the target mRNAs were calculated based on the comparative CT method [ΔΔCt (Cycle Threshold)].

**Mice functional phenotype.** We measured the clinical frailty index (CFI), Clinical Health Score (CHS), Physical Function Score (PFS), and Vitality Score in mice, as previously described (Marcozzi *et al.* 2023). All frailty measurements were performed in a dedicated INRCA SPF animal facility area. The CFI score for each mouse was calculated using a previously published checklist and method (Whitehead *et al.* 2014). The VS was obtained by calculating the arithmetic mean of the individual values of CHS and PFS.

PFS, an overall score representative of physical decline (Marcozzi *et al.* 2023), was computed as the mean of five composite scores related to physical frailty criteria: body size, strength, endurance, speed, and activity. Each score ranges from 1 (or slightly above excellent condition) to 0 (worse status).

The body size score is a composite score reflecting the body condition of the mice, including tumor-adjusted body weight and body length.

Strength Score is a composite score reflecting the mean of 4 different measurements of grip strength: Grip strength meter test (Ugo Basile, Varese, Italy) with a plastic grid, Grip strength meter test (Ugo Basile, Varese, Italy) with an iron bar, Home cage lift test and Gripping weights lift test (Malavolta *et al.* 2019).

Endurance Score was assessed through a composite score reflecting the endurance capacity of the mice that included treadmill distance (program: starting at 5 rpm for 2 min and increasing speed from 5 to 50 m/s in 2700 s), mean time to fall at rotarod test (program: starting at 5 m/s for 2 min and increasing speed from 5 to 40 rpm in 300 s), and the score of the gripping weights lift test normalized to body weight.

Speed Score was assessed through a composite score reflecting four different measurements related to the speed of the mice during their normal locomotion: highest speed interval that the mouse ran for at least 3 s in a locomotor activity test (5-min open field test on a 72 × 72 × 30 cm chamber), maximum speed of the rotarod test, mean of the measurements of the stride length of the mice obtained from a footprint test and video tracking.

**"*In vivo*" Bioluminescence assay.** Experimental animals ' bioluminescence imaging (BLI) was performed with an IVIS Spectrum system (PerkinElmer). Briefly, mice were intraperitoneally injected with 100 µl of Xenolight RediJect Coelenterazine h (PerkinElmer #760,506) and were immediately anesthetized in an oxygen-rich induction chamber with 2% isofluorane. Images were captured after 20 min of incubation to allow substrate distribution. Bioluminescent images were obtained with mice in the dorsal position. Anesthesia was maintained during imaging using a nose cone isofluorane-oxygen delivery device in the light-tight chamber. Bioluminescence, acquired by the CCD camera, was quantified by the Living Image® software, drawing a region of interest (ROI) around the interested area and measuring the radiance (photons/seconds/cm2/steradian) emitted by the surface within the ROI.

**Statistical analysis.** Generalized linear mixed model analysis (SPSS 26.0) was used to consider the longitudinal design of the pilot study in mice. The identifier of each mouse, gender, and age were included in the model. The linear models were developed assuming linear distribution with the identity link function. The Satterthwaite approximation with a robust estimator was used for unbalanced data and violation of the assumptions. Kaplan-Meier estimated differential survival patterns with the Log-Rank test. The chi-square test was used to test the distribution of pathologies detected by gross necroscopy performed during organ explants or post-mortem between treated and control groups.

The other statistical analyses were performed using the GraphPad Prism 10 software. Student's t, Wilcoxon, or Mann-Whitney U tests were used to compare normally, non-normally, or independent non-normally distributed data, respectively, between two experimental groups, ANOVA, and adequate *post hoc* tests for multiple comparisons. The General Linear Model with a univariate approach was employed to adjust for sex as a covariate. Data indicate the mean values of at least three independent experiments ± SD or SEM or the mean with 95% CI; sample sizes (n) and p values were reported in the corresponding figure legends. Outliers were identified and excluded using the ROUT method (Q = 1%) or Grubbs test (alpha = 0.05).

**Bibliography**

Aiello A, Bacci L, Re A, Ripoli C, Pierconti F, Pinto F, Masetti R, Grassi C, Gaetano C, Bassi PF, Pontecorvi A, Nanni S & Farsetti A (2016) MALAT1 and HOTAIR Long Non-Coding RNAs Play Opposite Role in Estrogen-Mediated Transcriptional Regulation in Prostate Cancer Cells. *Sci Rep* 6.

Baell JB & Nissink JWM (2018) Seven Year Itch: Pan-Assay Interference Compounds (PAINS) in 2017—Utility and Limitations. *ACS Chem Biol* 13, 36–44.

Bankhead P, Loughrey MB, Fernández JA, Dombrowski Y, McArt DG, Dunne PD, McQuaid S, Gray RT, Murray LJ, Coleman HG, James JA, Salto-Tellez M & Hamilton PW (2017) QuPath: Open source software for digital pathology image analysis. *Sci Rep* 7.

Banks JL, Beard HS, Cao Y, Cho AE, Damm W, Farid R, Felts AK, Halgren TA, Mainz DT, Maple JR, Murphy R, Philipp DM, Repasky MP, Zhang LY, Berne BJ, Friesner RA, Gallicchio E & Levy RM (2005) Integrated Modeling Program, Applied Chemical Theory (IMPACT). *J Comput Chem* 26, 1752–1780.

De Donato M, Righino B, Filippetti F, Battaglia A, Petrillo M, Pirolli D, Scambia G, De Rosa MC & Gallo D (2018) Identification and antitumor activity of a novel inhibitor of the NIMA-related kinase NEK6. *Sci Rep* 8.

Fuhrmann-Stroissnigg H, Ling YY, Zhao J, McGowan SJ, Zhu Y, Brooks RW, Grassi D, Gregg SQ, Stripay JL, Dorronsoro A, Corbo L, Tang P, Bukata C, Ring N, Giacca M, Li X, Tchkonia T, Kirkland JL, Niedernhofer LJ & Robbins PD (2017) Identification of HSP90 inhibitors as a novel class of senolytics. *Nat Commun* 8–422.

Gaulton A, Bellis LJ, Bento AP, Chambers J, Davies M, Hersey A, Light Y, McGlinchey S, Michalovich D, Al-Lazikani B & Overington JP (2012) ChEMBL: A large-scale bioactivity database for drug discovery. *Nucleic Acids Res* 40.

Itahana K, Campisi J & Dimri GP (2007) Methods to detect biomarkers of cellular senescence: The senescence-associated β-galactosidase assay. *Methods in Molecular Biology* 371, 21–31.

Jafari R, Almqvist H, Axelsson H, Ignatushchenko M, Lundbäck T, Nordlund P & Molina DM (2014) The cellular thermal shift assay for evaluating drug target interactions in cells. *Nat Protoc* 9, 2100–2122.

Lee YH, Kang BS & Bae YS (2014) Premature senescence in human breast cancer and colon cancer cells by tamoxifen-mediated reactive oxygen species generation. *Life Sci* 97, 116–122.

Li J, Abel R, Zhu K, Cao Y, Zhao S & Friesner RA (2011) The VSGB 2.0 model: A next generation energy model for high resolution protein structure modeling. *Proteins: Structure, Function and Bioinformatics* 79, 2794–2812.

Lipinski CA, Lombardo F, Lipinski CA, Dominy BW & Feeney PJ (2001) Experimental and computational approaches to estimate solubility and permeability in drug discovery and development settings. *Adv Drug Deliv Rev* 23, 3–26. Available at: https://www.researchgate.net/publication/246137377.

Malavolta M, Dato S, Villa F, Rango F De, Iannone F, Ferrario A, Maciag A, Ciaglia E, D'amato A, Carrizzo A, Basso A, Orlando F, Provinciali M, Madeddu P, Passarino G, Vecchione C, Rose G & Puca AA (2019) LAV-BPIFB4 associates with reduced frailty in humans and its transfer prevents frailty progression in old mice. *Aging* 11, 6555–6568.

Malavolta M, Giacconi R, Piacenza F, Strizzi S, Cardelli M, Bigossi G, Marcozzi S, Tiano L, Marcheggiani F, Matacchione G, Giuliani A, Olivieri F, Crivellari I, Beltrami AP, Serra A, Demaria M & Provinciali M (2022) Simple Detection of Unstained Live Senescent Cells with Imaging Flow Cytometry. *Cells* 11. Available at: https://www.mdpi.com/2073-4409/11/16/2506.

Marcozzi S, Bigossi G, Giuliani ME, Giacconi R, Cardelli M, Piacenza F, Orlando F, Segala A, Valerio A, Nisoli E, Brunetti D, Puca A, Boschi F, Gaetano C, Mongelli A, Lattanzio F, Provinciali M & Malavolta M (2023) Comprehensive longitudinal non-invasive quantification of healthspan and frailty in a large cohort (n = 546) of geriatric C57BL/6 J mice. *Geroscience* 45, 2195–2211.

Mysinger MM, Carchia M, Irwin JJ & Shoichet BK (2012) Directory of useful decoys, enhanced (DUD-E): Better ligands and decoys for better benchmarking. *J Med Chem* 55, 6582–6594.

Stebbins CE, Russo AA, Schneider C & Rosen N (1997) *Crystal Structure of an Hsp90-Geldanamycin Complex: Targeting of a Protein Chaperone by an Antitumor Agent The antitumor effects of Geldanamycin likely result from its ability to deplete cells of two broad classes of*,

Whitehead JC, Hildebrand BA, Sun M, Rockwood MR, Rose RA, Rockwood K & Howlett SE (2014) A clinical frailty index in aging mice: Comparisons with frailty index data in humans. *Journals of Gerontology - Series A Biological Sciences and Medical Sciences* 69, 621–632.

Wolber G, Dornhofer AA & Langer T (2006) Efficient overlay of small organic molecules using 3D pharmacophores. *J Comput Aided Mol Des* 20, 773–788.
